# Detection for disease tipping points by landscape dynamic network biomarkers

**DOI:** 10.1093/nsr/nwy162

**Published:** 2018-12-28

**Authors:** Xiaoping Liu, Xiao Chang, Siyang Leng, Hui Tang, Kazuyuki Aihara, Luonan Chen

**Affiliations:** 1 Key Laboratory of Systems Biology, Center for Excellence in Molecular Cell Science, Institute of Biochemistry and Cell Biology, Shanghai Institutes for Biological Sciences, Chinese Academy of Sciences, Shanghai 200031, China; 2 School of Mathematics and Statistics, Shandong University at Weihai, Weihai 264209, China; 3 Institute of Industrial Science, the University of Tokyo, Tokyo 153–8505, Japan; 4 Institute of Statistics and Applied Mathematics, Anhui University of Finance & Economics, Bengbu 233030, China; 5 School of Life Science and Technology, ShanghaiTech University, Shanghai 201210, China; 6 Center for Excellence in Animal Evolution and Genetics, Kunming 650223, China; 7 Research Center for Brain Science and Brain-Inspired Intelligence, Shanghai 201210, China

**Keywords:** single-sample network, dynamic network biomarkers, tipping points of complex disease

## Abstract

A new model-free method has been developed and termed the landscape dynamic network biomarker (*l*-DNB) methodology. The method is based on bifurcation theory, which can identify tipping points prior to serious disease deterioration using only single-sample omics data. Here, we show that *l*-DNB provides early-warning signals of disease deterioration on a single-sample basis and also detects critical genes or network biomarkers (i.e. DNB members) that promote the transition from normal to disease states. As a case study, *l*-DNB was used to predict severe influenza symptoms prior to the actual symptomatic appearance in influenza virus infections. The *l*-DNB approach was then also applied to three tumor disease datasets from the TCGA and was used to detect critical stages prior to tumor deterioration using an individual DNB for each patient. The individual DNBs were further used as individual biomarkers in the analysis of physiological data, which led to the identification of two biomarker types that were surprisingly effective in predicting the prognosis of tumors. The biomarkers can be considered as common biomarkers for cancer, wherein one indicates a poor prognosis and the other indicates a good prognosis.

## INTRODUCTION

**Figure 1.**
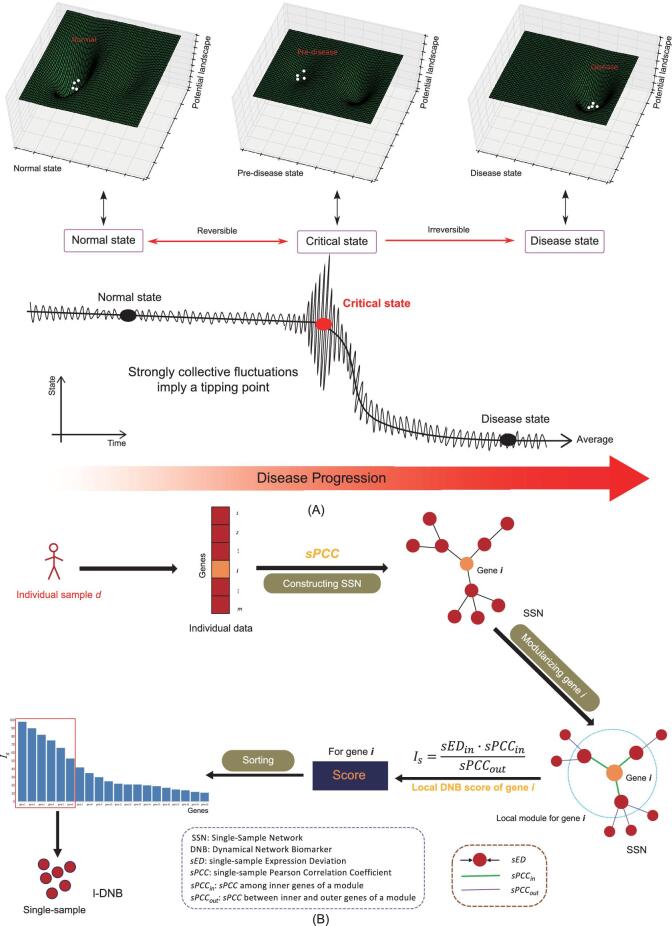
(A) Schematic diagram for disease progression of a complex disease in a subject. There are three states during disease progression comprising a normal state, a critical state (or pre-disease state or tipping point) and the final disease state. Generally, the phenotypic and molecular expressions of the disease state are significantly different from those of the normal state, but there are no significant differences observed between the critical and the normal states. Thus, detection of the critical state is difficult. However, there are strong collective fluctuations in processes at the critical state, which differs from the other states. (B) The *l*-DNB flowchart for identifying DNBs from a single sample. The individual data of sample *d* are used to construct the SSN for sample *d*. For every gene *x*, its local module comprises gene *x* and its first-order neighbors. The local module score for gene *x* can indicate the local DNB score for the gene. After ranking the scores for all genes, the top-*k* genes can be regarded as the potential DNB for the sample *d*. The *sED_n_, sPCC_in_* and *sPCC_out_* values for each individual sample are defined in the Methods.

Disease progression is a dynamic process that typically occurs non-linearly from a normal state via the gradual accumulation of small or quantitative changes that eventually result in a drastic or qualitative phenotypic transition to a disease state. Considerable evidence indicates the presence of critical states, or tipping points, just prior to the drastic transition between normal and disease states for many diseases [1–3]. The tipping point (or pre-disease state) during disease progression is the critical state, wherein reversion to the normal state is still possible and predictive information can be gathered for early-warning signals of imminent disease states (Fig. 1A). The identification of a tipping point for pre-cancerous states has recently garnered considerable attention [4]. However, identifying the critical state is difficult due to phenotypic and molecular expression similarities to the normal state that contrast with the stark differences observed between the disease and normal states. In particular, there are generally no significant differences in these properties between normal and critical states (Fig. 1A) [1–3]. Most traditional biomarkers of disease states are identified based on the differential expression of molecules between disease and normal states, rather than diagnosing critical states. Therefore, identifying tipping points or pre-disease states is an important challenge in medicine or biology that informs understanding of early-warning signals for the prevention and preemptive treatments of diseases in addition to the molecular mechanisms of complex diseases at a network level [1–3]. In particular, dynamic network biomarkers (DNBs) have been proposed to detect the critical states of many diseases using non-linear dynamic theory [1–3,5–7].

DNBs are a group of molecules (i.e. genes or proteins) or a molecule module that can signal the presence of a tipping point or critical state just prior to the drastic deterioration associated with complex diseases. There are three necessary prerequisites to discern DNB modules from data and identify tipping points (see Methods). In other words, if a physiological system approaches a critical state, then a DNB module appears and satisfies the three criticality conditions shown in Methods. The appearance of such a DNB module implies the imminent transition from a normal state to a disease state. The DNB module [1–3] can be obtained by maximizing the DNB score, *I_DNB_* in Equation (1):

(1) \begin{equation*}{I_{DNB}} = S{D_{in}}\frac{{PC{C_{in}}}}{{PC{C_{out}}}},\end{equation*}

where *SD_in_* is the standard deviation of gene expression in the DNB module, *PCC_in_* is the average of absolute Pearson correlation coefficients among genes in the DNB module and *PCC_out_* is the average of absolute Pearson correlation coefficients among genes compared within and outside of the DNB module.

The three components in Equation (1) represent the three criteria for determining criticality (see Methods) during disease progression [1–3,5,6]. Generally speaking, strongly collective fluctuations of a group of variables imply the presence of an imminent transition (Fig. 1A). The DNB method described above has been applied in the analysis of complex diseases and physiological processes by several research groups [1–3,5–8]. However, the DNB method requires multiple samples, which are generally unavailable for individual patients in clinical practices. In addition, despite the solid theoretical basis for the DNB methodology that is derived from non-linear dynamical theory and statistics, there are problems with computationally detecting DNB members and determining DNB module size, further limiting the application of the method. Here, a new method is proposed, termed the landscape DNB (*l-*DNB), that can be used to detect critical states of diseases via accurate and reliable identification of DNBs from a single sample. The method evaluates the local criticality or local DNB score *I_s_* using Equation (1) gene by gene and then compiles the overall local DNB scores into a landscape. The global criticality score, *I_DNB_*, can then be calculated from the landscape of the sample (or patient) by choosing those genes as DNB members with the highest local DNB scores.

Applying the *l*-DNB method to a dataset of influenza virus infection [9] as a case study, *l*-DNBs, as individual biomarkers, can reliably detect early-warning signals of disease state transition and accurately predict severe influenza symptoms in each individual at least 8 hours earlier than by conventional methods. The *l*-DNB method was further applied in the analysis of three different tumors, including lung adenocarcinoma (LUAD), kidney renal clear cell carcinoma (KIRC) and thyroid carcinoma (THCA). From these analyses, the critical states prior to the severe disease deterioration were identified among the different stages of the three tumors. In particular, the critical state for LUAD was identified in stage IIB, that of THCA in stage III and that of KIRC in stage II. In addition, we found that DNB members were effective in predicting prognoses when DNBs common across population samples were further applied to physiological data as common biomarkers. Moreover, the analyses indicated that DNB members can be categorized into two types of molecules to predict prognoses for the three tumors: one for samples with poor prognosis (i.e. pessimistic biomarkers) and another for samples with good prognosis (i.e. optimistic biomarkers). Taken together, these results indicate that *l*-DNB can reliably identify critical state of diseases using DNB modules on a single-sample basis. Importantly, the method quantifies early-warning signals before disease deterioration, and also provides real network biomarkers for disease prediction within each individual.

## RESULTS

### Identifying DNB modules in a single sample using the *l*-DNB method

Using *n* samples as the reference samples or data [10] that represent the expression data for *m* genes, the identification of DNBs within a single sample by *l-*DNB can be performed with the following three steps (Fig. 1B, Supplementary Fig. 19, available as Supplementary Data at *NSR* online, and Methods).

Step-1: Construction of a single-sample network (SSN) for the given sample (see Methods).

Step-2: Calculation of the local DNB score for every gene in the dataset. Using the three criteria for criticality [1,3,11], the local DNB score, *I_s_ (x)*, is calculated using Equation (8) (see Methods) for the local module centered at gene *x*, where the local module is gene *x* and its first-order network from the SSN [10], with *x* = 1, …, m.

Step-3: Identification of the DNB module for the single sample. Overall, local DNB scores are used to form a landscape, from which DNB genes (or the DNB module) and the global DNB score, *I_DNB_*, can be obtained for the sample or subject. Here, the DNB genes or DNB module are those genes with the highest-*k* local DNB scores, *I_s_(x)*, and the global DNB score, *I_DNB_*, is defined as

$$
\begin{equation*}
{I_{DNB}} = \sum\limits_{x = 1}^k {{I_s}(x)/k} .
\end{equation*}$$

The SSN can then be constructed by collecting the edges with significant *sPCC*s [10] for each sample (see Methods and Supplementary Fig. 1, available as Supplementary Data at *NSR* online). In each SSN, a gene and its first-order neighbors comprise a local module around the gene and the local DNB, *I_s_*, of the module is calculated using Equation (8) (Fig. 1B), where *x = 1, …, m*. Since a module has a central gene in the SSN, the module is considered the local module for this gene in the single sample (Fig. 1B). Hence, *I_s_* is used as the local DNB score for the local module centered at this gene in the single sample.

After the *I_s_(x)* of every gene *x* is obtained from its corresponding module in SSN, all of the genes are ranked in descending order by scores (Fig. 1B), which provides a landscape of local DNB scores (Supplementary Fig. 2, available as Supplementary Data at *NSR* online). The top-*k* genes in the ranked list can be regarded as the potential DNB members for the single sample and the corresponding global DNB score can be simply estimated by averaging *I_s_* over these top-*k* genes. Thus, the tipping point of the disease state can be quantified with the corresponding DNB in a reliable and systematic manner without clustering algorithms or other heuristic procedures (Supplementary Fig. 19, available as Supplementary Data at *NSR* online). The sample with the highest *I_DNB_* score among all of the samples is considered to be in the critical state or otherwise near the tipping point.

### Detecting early-warning signals of influenza virus infection with *l*-DNBs

The GSE30550 dataset comprises time series data for the influenza virus infection process [9] and was obtained from the GEO database to validate the detection of early-warning signals prior to disease onset using *l*-DNB (see Supplementary Methods, available as Supplementary Data at *NSR* online). Within the dataset, 17 healthy human volunteers were inoculated with H3N2 influenza virus and gene-expression profiles in host peripheral blood samples were examined at 16 time points for each individual (Supplementary Fig. 3, available as Supplementary Data at *NSR* online). Nine of the 17 volunteers (subjects) developed severe influenza symptoms, while the remaining 8 did not [9].

**Figure 2.**
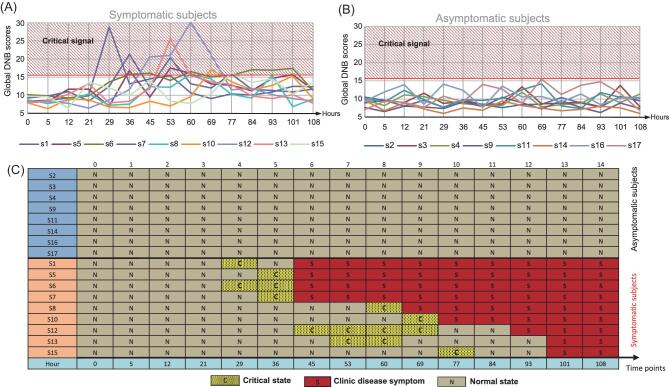
Identification of critical states for influenza viral infection data. (A) Line charts showing global DNB scores as early-warning signals in all symptomatic subjects; (B) line charts showing global DNB scores for all asymptomatic subjects; (C) *l-*DNB diagnoses and clinical diagnoses for all of the samples.

SSNs were constructed and the *I_s_* values were calculated for every gene (Fig. 1B) at each time point within each sample using reference data representing gene-expression data from all of the volunteers at the –24-hour time point (24 hours before viral inoculation, Supplementary Fig. 3, available as Supplementary Data at *NSR* online). The 20 highest-ranked genes by *I_s_* values were considered the DNB for each subject (i.e. individual biomarkers or individual DNBs for the sample) in each time point. Subsequently, the average *I_s_* value for the 20 top ranked genes (i.e. the *I_DNB_*) was defined as the global DNB score. The high score corresponds to an early-warning signal for disease state (Fig. 2). The *l*-DNB scores for the nine symptomatic subjects drastically increased before the appearance of influenza symptoms (Fig. 2A) and the scores for the eight non-symptomatic subjects remained stable and low throughout the experiment (Fig. 2B). Thus, the early-warning disease signals for the respective DNBs for influenza symptoms were detected in the nine symptomatic subjects at least 8 hours before the appearance of influenza symptoms, while no significant signal was observed in the eight non-symptomatic subjects (Fig. 2B and C). Note that the DNB genes of each subject were generally dissimilar (Supplementary Table 1, available as Supplementary Data at *NSR* online), indicating the presence of individual biomarkers for each subject rather than biomarkers that are common across all of the subjects. Enrichment analysis of high-frequency genes appearing in at least four DNBs (or subjects) indicated that the high-frequency genes were associated with some immunity-related biological processes annotated in the gene ontology database (http://www.ebi.ac.uk/QuickGO) including ‘immune response’ (GO:0006955), ‘regulation of lymphocyte mediated immunity’ (GO:0002706) and ‘regulation of adaptive immune response’ (GO:0002819). These observations are consistent with the process of viral infection. Critical states can certainly be detected in more than one time point for a sample, as was observed for volunteers s6, s12 and s13 (Fig. 2C). Similar results were obtained when using the 30 highest-ranked genes in the *I_s_* list as the DNB (Supplementary Fig. 4, available as Supplementary Data at *NSR* online), implying that *l*-DNBs can robustly identify pre-disease states and accurately detect early-warning signals for influenza viral infections. Therefore, the *l*-DNB method can be used to identify the critical states of diseases in a reliable and systematic manner using individual DNBs based on expression data from one sample taken in a clinic.

### Identifying critical states in tumors with *l*-DNB

**Figure 3.**
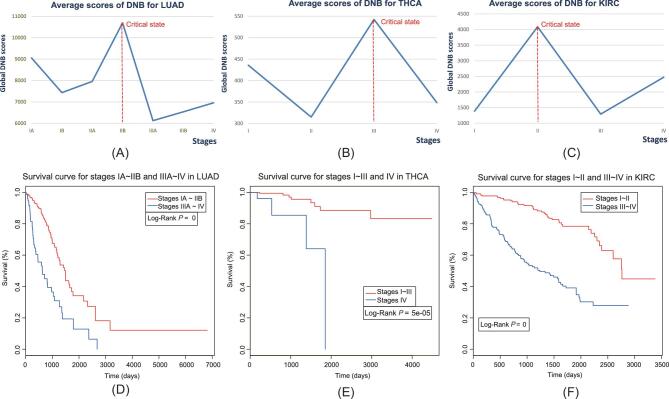
Identification of critical states for tumor deterioration in three cancers: (A) LUAD; (B) THCA; (C) KIRC. Comparison of survival curves before and after critical state for three cancers: (D) LUAD; (E) THCA; (F) KIRC.

The *l*-DNB methodology was further applied to investigate three tumor-associated gene-expression data sets for LUAD, KIRC and THCA that were obtained from the TCGA database (see Supplementary Methods, available as Supplementary Data at *NSR* online). SSNs were constructed for every tumor sample and the *I_s_* values for every gene were also calculated and ranked for each. The 20 highest-ranked genes in the *I_s_* list were chosen as the DNB module for each sample and global DNB scores, *I_DNB_*, were obtained for every tumor sample. The reference samples for each tumor were taken from tumor-adjacent samples and are described in further detail within the Supplementary Methods, available as Supplementary Data at *NSR* online. The average DNB scores were calculated for samples in each tumor stage against the respective reference samples and the resultant score curves are shown in Fig. 3A–C. According to DNB theory, the tipping point suggested by the DNB score is the critical state during disease progression [1–3,11]. The tipping point for LUAD was identified in stage IIB (Fig. 3A), after which the tumor state significantly deteriorated in patients, with LUAD-like disease phase transitions. Survival analysis was evaluated from the obtained DNBs for LUAD samples and compared against survival curves for samples before and after the critical state (stage IIB) using log-rank tests. The survival curves before and after stage IIB in LUAD samples were significantly different (*p* ∼ 0) (Fig. 3D). Moreover, the survival times of samples after the critical state were significantly shorter than for samples before the critical state (Fig. 3D). There were no significant differences in survival curves among samples in stages IA, IB and IIA (the stages prior to the critical state) (*P* = 0.7561; Supplementary Fig. 6a, available as Supplementary Data at *NSR* online). In addition, there were also no significant differences in survival curves among samples in stages IIIA, IIIB and IV (the stages after the critical state) (*P* = 0.47515; Supplementary Fig. 6b, available as Supplementary Data at *NSR* online). The survival times of samples in stage IIIA were significantly shorter than for samples in stage IIB (*P* = 0.0087; Supplementary Fig. 7, available as Supplementary Data at *NSR* online). These results indicate the sudden deterioration of survival times in patients with LUAD after the IIB stage, strongly implying that the IIB stage is the disease state tipping point.

*l*-DNB analyses also identified the tipping point/critical state for THCA disease states as stage III (Fig. 3B), after which significant tumor deterioration occurred. The survival curves for samples before and after the critical state of THCA were significantly different (*P* = 5 × 10^−5^; Fig. 3E). Accordingly, the survival times of samples after the critical state were significantly shorter than for samples prior to the critical state (Fig. 3E). In addition, the critical state/tipping point for tumor deterioration in KIRC was identified in stage II (Fig. 3C). The survival curves for samples before and after the KIRC critical state were significantly different (*P* ∼ 0; Fig. 3F). Accordingly, survival times of samples after the critical state were significantly shorter than for samples before the critical state (Fig. 3F). Taken together, the results provided here demonstrate that *l*-DNB can identify the critical states associated with disease deterioration in cancers.

### Prognostic prediction of tumors using *l*-DNB

In addition to identifying critical states, *l*-DNB is effective in predicting prognoses. Indeed, DNB members could be categorized into two types of molecules for prognostic prediction as common biomarkers for all of the samples: those for samples with poor prognosis, termed pessimistic biomarkers; and those with good prognosis, termed optimistic biomarkers. Additional details for these identifications are provided in the Supplementary Methods, available as Supplementary Data at *NSR* online.

If pessimistic biomarkers appeared in a sample's DNB, then the prognosis for the sample would be more pessimistic than for other samples. Likewise, if optimistic biomarkers were detected in the DNB of a sample, the prognosis for the sample would be more optimistic than for others.

**Figure 4.**
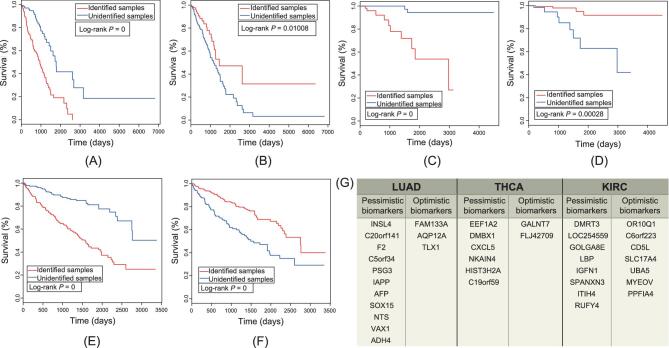
Comparison of survival curves for pessimistic and optimistic biomarkers identified for three cancers: (A) pessimistic biomarkers for LUAD; (B) optimistic biomarkers for LUAD; (C) pessimistic biomarkers for THCA; (D) optimistic biomarkers for THCA; (E) pessimistic biomarkers for KIRC; (F) optimistic biomarkers for KIRC. Pessimistic or optimistic biomarkers in the DNB are indicated by ‘Identified sample’, while ‘Unidentified sample’ refers to other samples. (G) Genes representing pessimistic and optimistic biomarkers for LUAD, THCA and KIRC disease states.

A total of 11 genes were identified as pessimistic biomarkers for LUAD (Fig. 4G and Supplementary Fig. 8, available as Supplementary Data at *NSR* online), while 3 were identified as optimistic biomarkers (Fig. 4G and Supplementary Fig. 9, available as Supplementary Data at *NSR* online). The survival times of the samples identified with pessimistic biomarkers for LUAD were significantly shorter than for other samples (*P* ∼ 0; Fig. 4A). Further, significantly longer survival times were observed for samples identified with optimistic biomarkers for LUAD than for other samples (*P* = 0.01008; Fig. 4B). Thus, if one or more pessimistic biomarker genes were present in the DNB of the subject's sample, they would have a shorter survival time, while the converse was true for those samples with optimistic biomarkers.

To test the predictive power of DNB members, we separated samples identified with pessimistic biomarkers into three groups. The three groups exhibited with only one, at least two and at least three pessimistic biomarkers in their DNBs, respectively. The survival times of group-2 were shorter than those for group-1 (Supplementary Fig. 10a, available as Supplementary Data at *NSR* online), although the difference was not statistically significant (*P* = 0.0513). In addition, the survival times for group-3 were shorter than those of group-1 (Supplementary Fig. 10b, available as Supplementary Data at *NSR* online) and this difference was statistically significant (*P* = 0.00768). These results indicate that, when more pessimistic biomarker genes were present in the DNB of a patient, shorter survival times would be expected for the patient. Hence, the pessimistic and optimistic biomarkers for LUAD can be used to provide accurate prognosis or predict survival times in patients with LUAD.

In addition, six pessimistic biomarker genes (Fig. 4G and Supplementary Fig. 11, available as Supplementary Data at *NSR* online) and two optimistic biomarker genes (Fig. 4G and Supplementary Fig. 12, available as Supplementary Data at *NSR* online) were identified for THCA. The survival times of samples identified with pessimistic biomarkers for THCA were significantly shorter than those for other samples (*P* ∼ 0; Fig. 4C). Conversely, significantly longer survival times were observed for samples identified with optimistic biomarkers for THCA than for other samples (*P* = 0.00028; Fig. 4D). Thus, the pessimistic and optimistic biomarkers for THCA can be used to make accurate prognoses and estimate survival times for patients with THCA.

Lastly, eight pessimistic biomarker genes (Fig. 4G and Supplementary Fig. 13, available as Supplementary Data at *NSR* online) and seven optimistic biomarker genes (Fig. 4G and Supplementary Fig. 14, available as Supplementary Data at *NSR* online) were identified for KIRC. As in the above analyses, pessimistic biomarkers for KIRC can be used to identify potentially shorter survival times in patients with KIRC, while optimistic biomarkers for KIRC can be used to identify potentially longer survival times. The samples with pessimistic biomarkers exhibited shorter survival times than did other samples (*P* ∼ 0; Fig. 4E) and samples with optimistic biomarkers exhibited longer survival times than did other samples (*P* ∼ 0; Fig. 4F).

## DISCUSSION

The *l*-DNB methodology described here represents a novel approach to reliably and accurately identify critical states and detect early-warning signals of complex diseases on a single-sample basis. In this study, the *l*-DNB algorithm was developed to identify individual DNBs from single samples and the ability of *l-*DNB to detect early-warning signals for four different diseases, namely influenza, LUAD, THCA and KIRC, was validated. The *l*-DNB approach described here can be used to detect local criticality score or local DNB score for every gene within a sample, and the 20–50 top ranked genes are empirically considered as the suitable DNB size in a sample. Consequently, DNB can be robustly obtained using *l*-DNB for any single sample, wherein higher DNB scores indicate a higher risk for health deterioration or an imminent disease status for the subject.

Application of individual DNBs to clinical data indicated that DNB members are effective for prognostic analyses, as evinced by the identification of pessimistic and optimistic biomarkers for LUAD, THCA and KIRC disease states that can be used to evaluate patient prognosis for each of the three diseases. Accordingly, if a patient's DNB included pessimistic biomarkers, the patient was likely to have shorter survival times. Moreover, greater numbers of pessimistic biomarkers in patient DNBs correlated with shorter patient survival times. Conversely, if a patient's DNB included optimistic biomarkers, the patient would be expected to have longer survival times. Importantly, some biomarkers indicated in Fig. 4G have been previously associated with their corresponding tumors. For example, down-regulation of insulin-like growth factor 4 (INSL4) appeared to contribute to a slower growth rate and loss of tumorigenic properties in a cell line of LUAD [12]. Likewise, down-regulation of Alpha-fetoprotein (AFP) has been reported to result in LUAD [13,14]. Some of the biomarkers identified here were related to other cancers and have not been previously reported to be associated with LUAD, THCA or KIRC. For example, SOX15 is related to pancreatic tumors, esophagus tumors and embryonal cell carcinoma [15–17], while CXCL5 is related to laryngeal cancer and glioma [18,19] and TLX1 is related to leukemia [20,21]. Nevertheless, most of the biomarkers indicated in Fig. 4G have not been previously reported in association with any tumor disease state. Thus, the pessimistic and optimistic biomarkers identified here could be important targets for future research into the molecular mechanisms underlying tumor onset or disease state deterioration. Four genes (*PSG3, AFP* and *ADH4* in LUAD, in addition to *SPANXN3* in KIRC) were identified as pessimistic biomarkers, but were actually not differentially expressed between the identified and unidentified samples (Supplementary Table 2, available as Supplementary Data at *NSR* online), and thus they cannot be detected by traditional methods that rely on differential expression patterns. This result implies that the *l*-DNB method can reveal ‘Dark Matter’ [22,23] genes (i.e. those without differential expressions) that are usually ignored by traditional analyses.

Importantly, the method proposed here is model-free and does not require learning processes to identify biomarkers, which represents an advantage over traditional classification or machine learning methods that require a large number of case/control samples for supervised or unsupervised learning, in order to avoid overlearning issues. Specifically, the *l*-DNB method is constructed from three model-free DNB conditions for each sample that are based on the essential dynamical features of critical states for general biological systems. Consequently, the method inherently identifies individual biomarkers rather than common biomarkers without overlearning problems. It should be noted, however, that the identification of common biomarkers or identifying a common threshold of criticality across all of the individuals for each disease may require data for whole populations.

The *l-*DNB method is also robust with respect to reference samples owing to the mechanisms underlying SSN construction, which has been discussed in detail previously [10]. Specifically, similar *l-*DNB rankings can be obtained from SSNs for a single sample with different reference samples. Thus, similar *l*-DNB rankings can be obtained from different samples with similar network structures. Moreover, the differential network (differential Pearson correlation coefficient (PCC)) between any two samples eliminates common components, including those of the reference samples, thereby further reducing the influence of reference samples on outcomes. However, when reference sample sizes are small (e.g. <10), they may significantly impact rankings. Additional details of validation from other independent datasets and other SSN methods are provided in the Supplementary Information, available as Supplementary Data at *NSR* online.

## CONCLUSION

The *l*-DNB method proposed here can quantify early-warning signals for disease states on a single-sample basis prior to disease onset or disease states deterioration. Moreover, the method can be used to identify effective network biomarkers that can predict the prognosis of each cancer patient. Consequently, the methodology has great potential for direct application in preventive and personalized medicine [24–27]. Specifically, the *l*-DNB method can detect DNBs systematically without clustering algorithms or other heuristic procedures that have previously been used. Hence, it can be directly applied to personalized pre-disease diagnosis and also the analysis of molecular mechanisms associated with disease progression at the network level. Similarly, the *l-*DNB method could also be used to detect critical states of many non-linear biological processes, including cellular differentiation and cellular proliferation [1–3,5–6].

## METHODS

### The three criteria for DNB identification

The DNB theory is provided in Supplementary Methods, available as Supplementary Data at *NSR* online, and is based on the premise that, when a biological or physiological system approaches a critical state from a stable normal state, a DNB module or a group of molecules (i.e. variables) appears and satisfies the following three statistical conditions [2,4]:

[Condition 1] deviation for each molecule inside the module (*SD_in_*: standard deviation) drastically increases;
[Condition 2] correlation between molecules inside the module (*PCC_in_*: Pearson correlation coefficients in absolute values) rapidly increase; and
[Condition 3] correlation between molecules inside and outside of the modules (*PCC_out_*: Pearson correlation coefficients in absolute values) rapidly decrease.

### Construction of SSNs

Given *n* reference samples, the PCC between genes *x* and *y* in the reference sample data can be calculated as

(2) \begin{equation*}PC{C_n}(x,y) = \frac{{\sum\limits_{i = 1}^n {({x_i} - \bar{x})({y_i} - \bar{y})} }}{{\sqrt {\sum\limits_{i = 1}^n {{{({x_i} - \bar{x})}^2}\sum\limits_{i = 1}^n {{{({y_i} - \bar{y})}^2}} } } }},\end{equation*}

where *x_i_* and *y_i_* are the expression values for genes *x* and *y* for the *i*th sample in the reference samples, respectively. $\bar{x}$ and $\bar{y}$ are the average gene-expression values of genes *x* and *y* in the reference samples, respectively.

*PCC_n_*(*x, y*) is the correlation between genes *x* and *y* in the *n* reference samples. After a new sample is added to the reference sample set (Supplementary Fig. 1, available as Supplementary Data at *NSR* online), a new *PCC* is calculated for the two genes using Equation (2) based on the total *n*+1 samples (i.e. *n* reference samples and the one new sample *d*) as *PCC_n_*_+1_(*x, y*). The difference between *PCC_n+1_* and *PCC_n_* for the two genes is due to the new sample addition to the reference data (Supplementary Fig. 1, available as Supplementary Data at *NSR* online) and hence characterizes the correlation between this sample and the *n* reference samples. Thus, the single-sample *PCC* (*sPCC*) of the two genes *x* and *y* against the *n* reference sample is defined as follows [10]:

(3) \begin{equation*}sPC{C_n}(x,y) = PC{C_{n + 1}}(x,y) - PC{C_n}(x,y),\end{equation*}

which actually represents a differential *PCC* or a perturbed *PCC* of this sample against the reference samples. Since *PCCs* follow a *t*-distribution, *sPCC_n_* in Equation (3) follows the differential PCC distribution with consecutive samples. The statistical significance of *sPCC_n_* can be accurately evaluated using the volcano distribution derived from the distribution of the *n* reference samples or via SSN theory [10]. Thus, the distribution of *sPCC_n_* depends on both the *PCC_n_* and *n* values.

To reduce computational complexity, an approximation scheme can be used to estimate the significance of *sPCC_n_* of Equation (3). Specifically, by assuming a Gaussian distribution with a sufficiently large *n*, a ‘Z’ score can be calculated for each *sPCC* and the *p*-value of each *sPCC* can be approximated from the standard normal cumulative distribution [28] based on the ‘Z’ scores [10].

SSNs can then be identified based on significant *sPCC*s among all of the pairs of genes or molecules that are perturbed by the single sample, while this network also characterizes the single sample [10]. Further, the *sPCC_n_* for the SSN can be directly used as an approximation without significance tests. In addition to using the differential *sPCC* values of Equation (3), SSNs can also be constructed using correlation-like edge of each sample (Supplementary Methods, available as Supplementary Data at *NSR* online) [29,30].

### Estimating deviation in a single sample

Given *n* reference samples (i.e. the normal or control sample dataset), the distribution of each gene's expression can be obtained as its reference distribution. The expression of a gene in a new sample *d* (e.g. a case sample for statistical testing) can be compared with its reference distribution to estimate the deviation of its expression from the reference samples (*n* samples). The standard deviation of gene expression in the new sample can be expressed as the deviation from expectation based on its reference distribution (Supplementary Fig. 1, available as Supplementary Data at *NSR* online). According to Condition 1 of the DNB identification, the deviation of gene *x* expression in a single sample against its expression in the *n* reference samples, namely the single-sample Expression Deviation *(sED*), can be defined as:

(4) \begin{equation*}sED({x_d}) = \left| {{x_d} - \bar{x}} \right|,\end{equation*}

where *x_d_* is the expression of gene *x* in the new sample *d* and $\bar{x}$ is the average expression of gene *x* in the *n* reference samples, namely

$$\begin{equation*}\bar{x} = \frac{1}{n}\sum\limits_{i = 1}^n {{x_i}} .\end{equation*}$$

Hence, *sED* can be regarded as Condition 1 of DNB for the *l-*DNB method. Then, we have

(5) \begin{equation*}sE{D_{in}} = \frac{1}{{1 + {n_{{x_d}}}}}\Bigg[\! {sED({x_d}) +\!\!\! \sum\limits_{{y_d} \in {N_{{x_d}}}}\!\! {sED({y_d})} }\! \Bigg],\end{equation*}

which represents the average deviation in expression of all of the (1+*n_xd_*) genes in the local module of gene *x* for sample *d* relative to the reference samples. Here, the local module is gene *x* and its first-order neighbors, *N_xd_* (where *N_xd_* is a gene set with *n_xd_* genes), which are based on the SSN.

### Estimating correlation in a single sample

If *n* is sufficiently large, ∣*PCC_n_*∣ is considered to be larger than ∣*sPCC_n_*∣ (i.e. ∣*PCC_n_*∣ > ∣*sPCC_n_*∣). Here, *PCC_n_* of Equation (2) and *sPCC_n_* of Equation (3) are used to evaluate Conditions 2 and 3 and estimate local DNBs. Specifically, *PCC_in_*is proportional to *sPCC_in_* and is defined as

(6) \begin{equation*}sPC{C_{in}} = \frac{1}{{{n_{{x_d}}}}}\sum\limits_{{y_d} \in {N_{{x_d}}}} {|sPC{C_n}({x_d},{y_d})|} ,\end{equation*}

where *sPCC_in_* for the local module of gene *x* is the average value of *sPCC_n_* between gene *x* and its first-order neighbors, *N_xd_*, in the SSN. Thus, if the correlation for *PCC_n__+1_* (in absolute values) increases due to the additional sample *d* (Condition 2), then *sPCC_in_* increases.

*PCC_out_* (Condition 3) is defined as the average correlation between the inner and outer genes of the local DNB module for gene *x.* Since the neighbors of gene *x* may or may not be DNB members, s*PCC_out_* is heuristically defined as

(7) \begin{eqnarray*} \!\!\!\!\!\! sPC{C_{out}} = \frac{1}{{{n_{{x_d}}}{m_{{x_d}}}}}\!\sum\limits_{\begin{array}{l}\\{\scriptscriptstyle{x_d} \in {N_{{x_d}}}},\\ \scriptscriptstyle{{y_d} \in {M_{{x_d}}}}\end{array}}\!\! {{\rm{|}}sPC{C_n}({x_d},{y_d}){\rm{|}}} . \end{eqnarray*}

Here, *sPCC_out_* for the local module of gene *x* is proportional to the average value of *sPCC_n_* between the first-order neighbors, *N_xd_*, and the second-order neighbors, *M_xd_* (*M_xd_* is a gene set with *m_xd_* genes), for gene *x* in the SSN (Fig. 1B). Note that Equations (6) and (7) approximate *PCC_in_* and *PCC_out_* of the sample *d* and there may be other ways to effectively and accurately estimate these values.

### Calculation of local DNB scores for each gene in a single sample

The local DNB score, *I_s_(x)*, of each gene *(x = 1, 2, …, m)* can be obtained via Equation (8) for its local module, which is based on the SSN and the three statistical conditions for DNBs, similar to Equation (1):

(8) \begin{equation*}{I_s}(x) = sE{D_{in}}\frac{{sPC{C_{in}}}}{{sPC{C_{out}}}},\end{equation*}

where *I_s_(x)* is the score for the local module of gene *x* based on the single sample. In addition, the local DNB score *I_s_(x)* for each gene *x* can be considered as the criticality score for the gene and can thus be used to rank the importance of genes contributing to the criticality of the whole dynamic physiological system.

The detailed algorithm used to identify DNB modules and tipping points for a single sample are provided in the Supplementary Methods, available as Supplementary Data at *NSR* online.

## Supplementary Material

nwy162_Supplemental_Files
