# Supplementary material for: Detection for disease tipping points by landscape dynamic network biomarkers
Source: Natl Sci Rev. 2018 Dec 28;6(4):775–85. doi: 10.1093/nsr/nwy162 (PMC8291500; doi:10.1093/nsr/nwy162)
Supplement: nwy162_Supplemental_Files [file nwy162_supplemental_files.zip › Figure_S1.pdf]

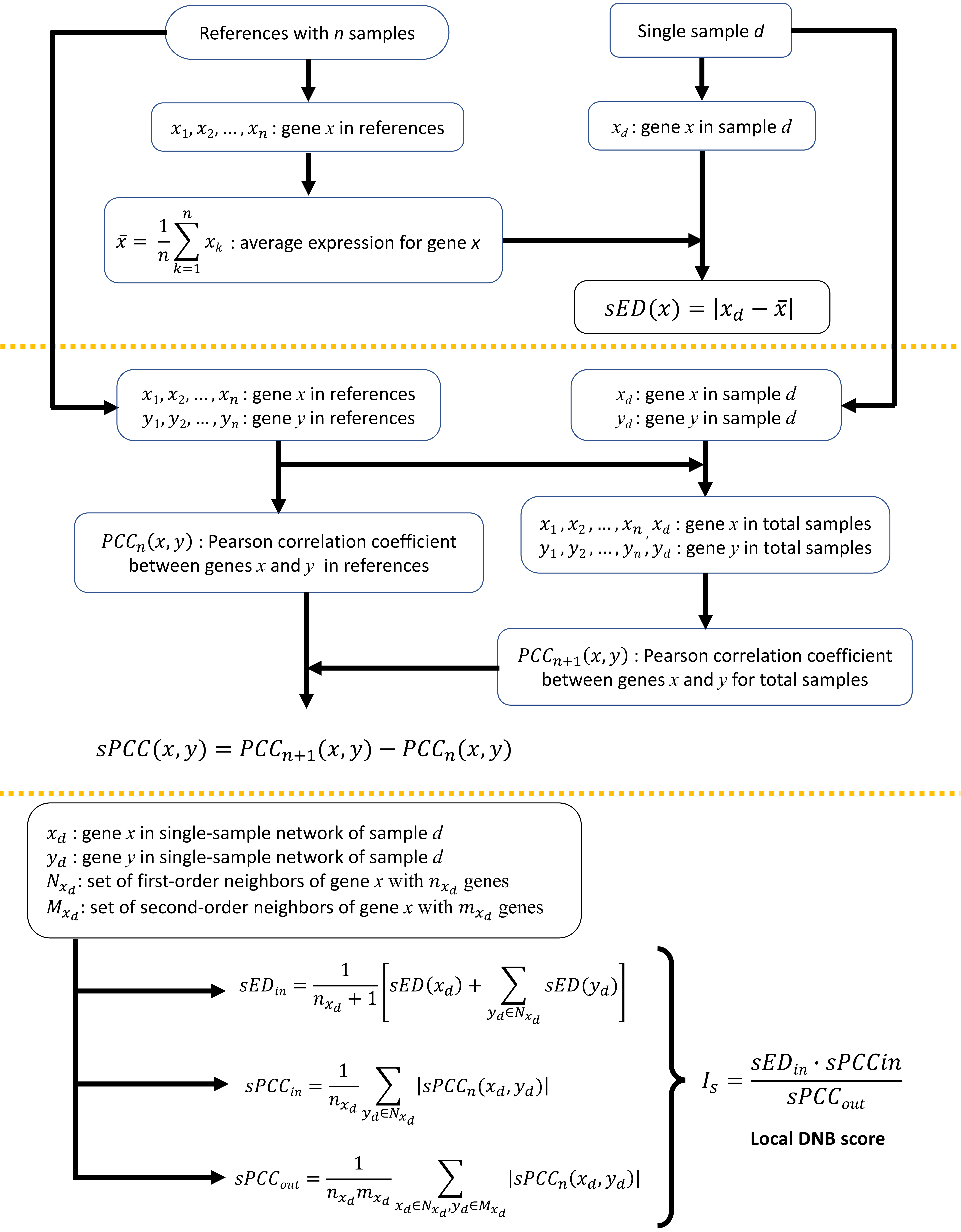

References with n samples
Single sample d
$x_1, x_2, \ldots, x_n$: gene x in references
x_d : gene x in sample d
$\bar{x} = \frac{1}{n}\sum_{k=1}^{n} x_k$: average expression for gene x
$sED(x) = |x_d - \bar{x}|$
$x_1, x_2, \ldots, x_n$: gene x in references
$y_1, y_2, \ldots, y_n$: gene y in references
x_d : gene x in sample d
y_d : gene y in sample d
$PCC_n(x, y)$: Pearson correlation coefficient between genes x and y in references
$x_1, x_2, \ldots, x_n, x_d$: gene x in total samples
$y_1, y_2, \ldots, y_n, y_d$: gene y in total samples
$PCC_{n+1}(x, y)$: Pearson correlation coefficient between genes x and y for total samples
$sPCC(x, y) = PCC_{n+1}(x, y) - PCC_n(x, y)$
x_d : gene x in single-sample network of sample d
y_d : gene y in single-sample network of sample d
N_{x_d}: set of first-order neighbors of gene x with n_{x_d} genes
M_{x_d}: set of second-order neighbors of gene x with m_{x_d} genes
$sED_{in} = \frac{1}{n_{x_d} + 1}\left[sED(x_d) + \sum_{y_d \in N_{x_d}} sED(y_d)\right]$
$sPCC_{in} = \frac{1}{n_{x_d}} \sum_{y_d \in N_{x_d}} |sPCC_n(x_d, y_d)|$
$sPCC_{out} = \frac{1}{n_{x_d} m_{x_d}} \sum_{x_d \in N_{x_d}, y_d \in M_{x_d}} |sPCC_n(x_d, y_d)|$
$I_s = \frac{sED_{in} \cdot sPCCin}{sPCC_{out}}$
Local DNB score
