# Supplementary material for: Detection for disease tipping points by landscape dynamic network biomarkers
Source: Natl Sci Rev. 2018 Dec 28;6(4):775–85. doi: 10.1093/nsr/nwy162 (PMC8291500; doi:10.1093/nsr/nwy162)
Supplement: nwy162_Supplemental_Files [file nwy162_supplemental_files.zip › Figure_S6.pdf]

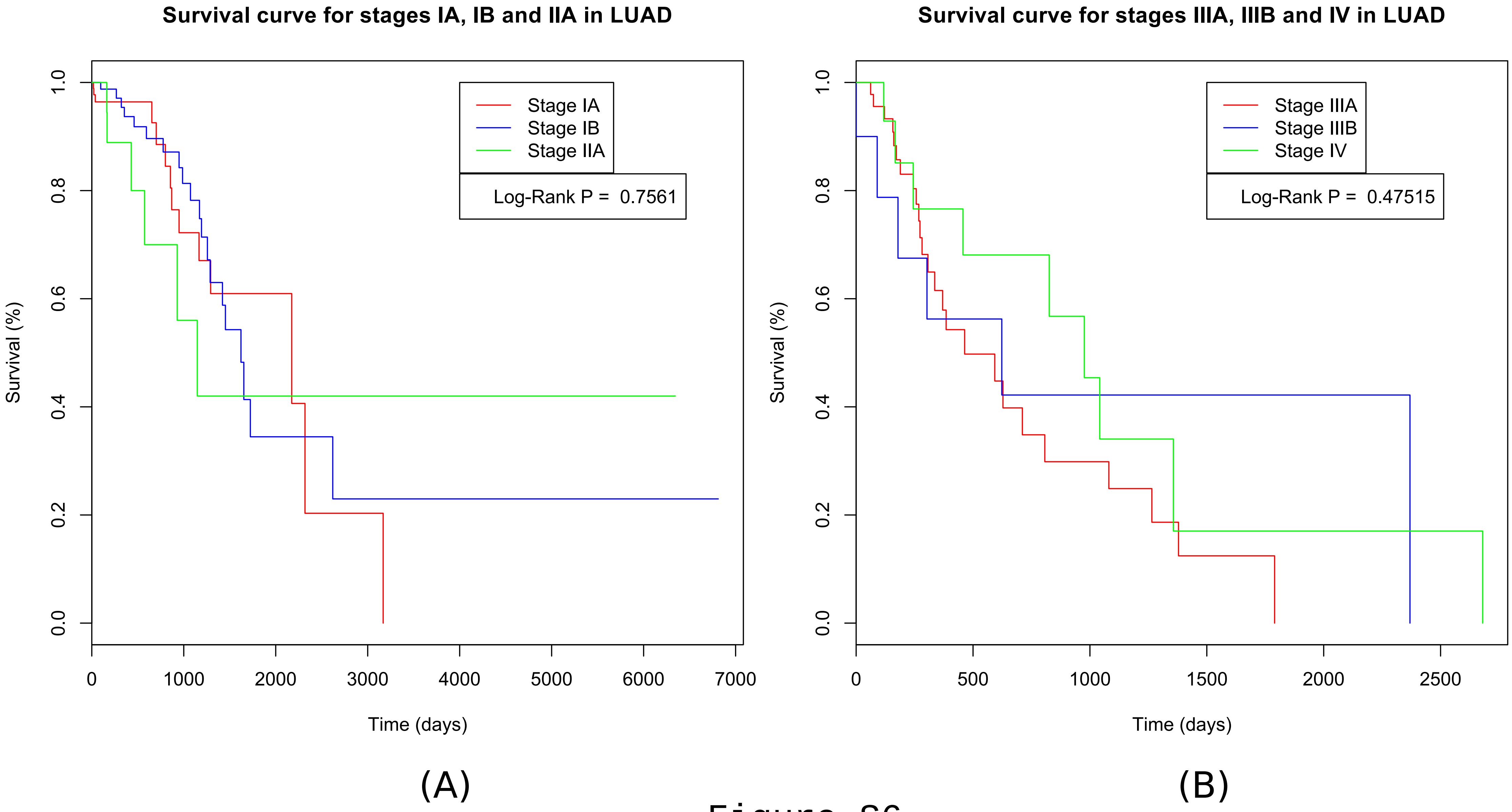
Survival curve for stages IA, IB and IIA in LUAD
Stage IA
Stage IB
Stage IIA
Log-Rank P = 0.7561
Survival (%)
Time (days)
0.0
0.2
0.4
0.6
0.8
1.0
0
1000
2000
3000
4000
5000
6000
7000
(A)
Survival curve for stages IIIA, IIIB and IV in LUAD
Stage IIIA
Stage IIIB
Stage IV
Log-Rank P = 0.47515
Survival (%)
Time (days)
0.0
0.2
0.4
0.6
0.8
1.0
0
500
1000
1500
2000
2500
(B)

Figure S6
