# Supplementary material for: Detection for disease tipping points by landscape dynamic network biomarkers
Source: Natl Sci Rev. 2018 Dec 28;6(4):775–85. doi: 10.1093/nsr/nwy162 (PMC8291500; doi:10.1093/nsr/nwy162)
Supplement: nwy162_Supplemental_Files [file nwy162_supplemental_files.zip › Table_S1.docx]

Table S1: the genes in the DNB for the symptomatic samples in influenza virus infection.

The table is an Excel file, please access it on below URL:

<https://github.com/xp-liu/Supplementary-Tables/blob/master/Table%20S1.xlsx?raw=true>

or

<https://sourceforge.net/projects/l-dnb/files/Table%20S1.xlsx/download>
