# Supplementary material for: Detection for disease tipping points by landscape dynamic network biomarkers
Source: Natl Sci Rev. 2018 Dec 28;6(4):775–85. doi: 10.1093/nsr/nwy162 (PMC8291500; doi:10.1093/nsr/nwy162)
Supplement: nwy162_Supplemental_Files [file nwy162_supplemental_files.zip › Table_S2.docx]

Table S2: The differential expressions for pessimistic biomarkers in LUAD, THCA, and KIRC between identified and unidentified samples.

| LUAD | | THCA | | KIRC | |
| --- | --- | --- | --- | --- | --- |
| **Pessimistic biomarkers** | ***P*-value of t test** | **Pessimistic biomarkers** | ***P*-value of t test** | **Pessimistic biomarkers** | ***P*-value of t test** |
| INSL4  C20orf141  F2  C5orf34  PSG3  IAPP  AFP  SOX15  NTS  VAX1  ADH4 | 7.337e-09  0.01383  0.03087  3.734e-07  0.13  0.0159  0.2919  1.040e-06  0.01098  0.0002428  0.05654 | EEF1A2  DMBX1  CXCL5  NKAIN4  HIST3H2A  C19orf59 | 5.034e-06  0.0008065  7.783e-12  0.03673  3.029e-05  6.581e-06 | DMRT3  LOC254559  GOLGA8E  LBP  IGFN1  SPANXN3  ITIH4  RUFY4 | 2.935e-06  7.120e-15  0.001684  7.182e-06  0.01502  0.07477  4.682e-09  8.054e-06 |

Red color: *p*-value > 0.05

Green color: 0.05 > *p*-value > 0.01

Other: *p*-value < 0.01
