# Supplementary material for: Detection for disease tipping points by landscape dynamic network biomarkers
Source: Natl Sci Rev. 2018 Dec 28;6(4):775–85. doi: 10.1093/nsr/nwy162 (PMC8291500; doi:10.1093/nsr/nwy162)
Supplement: nwy162_Supplemental_Files [file nwy162_supplemental_files.zip › Table_S3.docx]

Table S3: The number of tumor samples within each stage in the cancer datasets from TCGA

|  |  | **LUAD** | **KIRC** | **THCA** |
| --- | --- | --- | --- | --- |
| **Stage I** | **Stage IA** | 106 | 197 | 218 |
|  | **Stage IB** | 124 |  |  |
| **Stage II** | **Stage IIA** | 39 | 41 | 44 |
|  | **Stage IIB** | 59 |  |  |
| **Stage III** | **Stage IIIA** | 62 | 112 | 82 |
|  | **Stage IIIB** | 10 |  |  |
| **Stage IV** | **Stage IV** | 21 | 68 | 13 |
| **TA samples** | **TA samples** | 58 | 72 | 58 |

LUAD (lung adenocarcinoma), KIRC(kidney renal clear cell carcinoma), and THCA (thyroid carcinoma), TA samples are tumor-adjacent samples, and were used as the reference dataset in this study.
