# Supplementary material for: Detection for disease tipping points by landscape dynamic network biomarkers
Source: Natl Sci Rev. 2018 Dec 28;6(4):775–85. doi: 10.1093/nsr/nwy162 (PMC8291500; doi:10.1093/nsr/nwy162)
Supplement: nwy162_Supplemental_Files [file nwy162_supplemental_files.zip › Table_S4.docx]

Table S4: The stage distribution for the tumor samples of lung adenocarcinoma (LUAD), Kidney renal clear cell carcinoma (KIRC) and thyroid carcinoma (THCA) from TCGA

The table is an Excel file, please access it on below URL:

<https://github.com/xp-liu/Supplementary-Tables/blob/master/Table%20S4.xlsx?raw=true>

or

<https://sourceforge.net/projects/l-dnb/files/Table%20S4.xlsx/download>
