# Supplementary material for: Detection for disease tipping points by landscape dynamic network biomarkers
Source: Natl Sci Rev. 2018 Dec 28;6(4):775–85. doi: 10.1093/nsr/nwy162 (PMC8291500; doi:10.1093/nsr/nwy162)
Supplement: nwy162_Supplemental_Files [file nwy162_supplemental_files.zip › Table_S6.docx]

Table S6: The results of cross validation for the *l-DNB* method on influenza data.

|  | LOOCV | | | | LTOCV | | | | LThOCV | | | |
| --- | --- | --- | --- | --- | --- | --- | --- | --- | --- | --- | --- | --- |
| *k* | 20 | 25 | 30 | 35 | 20 | 25 | 30 | 35 | 20 | 25 | 30 | 35 |
| Accuracy | 1 | 0.941 | 0.941 | 0.941 | 0.989 | 0.941 | 0.945 | 0.938 | 0.985 | 0.958 | 0.963 | 0.956 |
| False positive rate | 0 | 0 | 0 | 0 | 0 | 0 | 0 | 0.0074 | 0 | 0 | 0 | 0.0082 |
| False negative rate | 0 | 0.0588 | 0.0588 | 0.0588 | 0.011 | 0.059 | 0.055 | 0.055 | 0.015 | 0.042 | 0.037 | 0.0359 |

LOOCV: Leave one out cross validation

LTOCV: Leave two out cross validation

LThOCV: Leave three out cross validation
