# Supplementary figures and images for: Detection for disease tipping points by landscape dynamic network biomarkers
Source: Natl Sci Rev. 2018 Dec 28;6(4):775–85. doi: 10.1093/nsr/nwy162 (PMC8291500; doi:10.1093/nsr/nwy162)

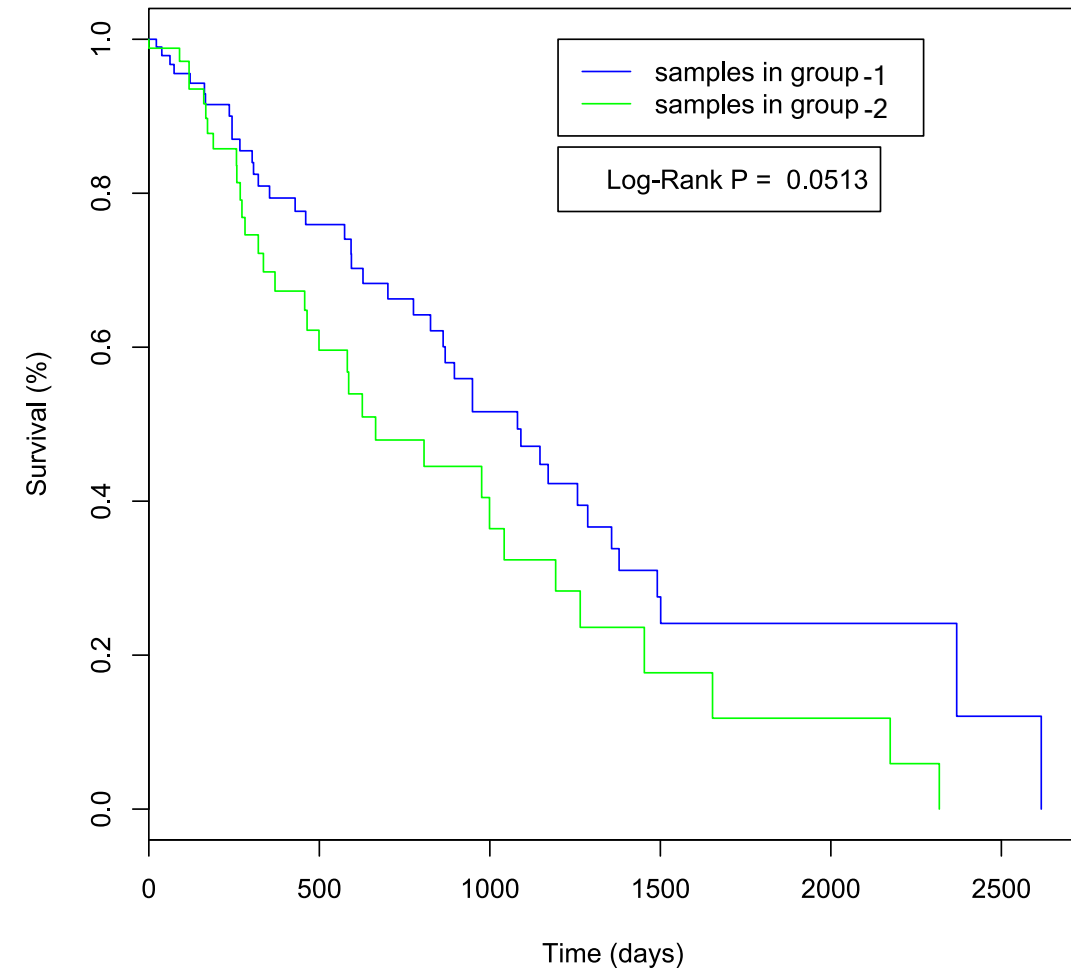

(A)

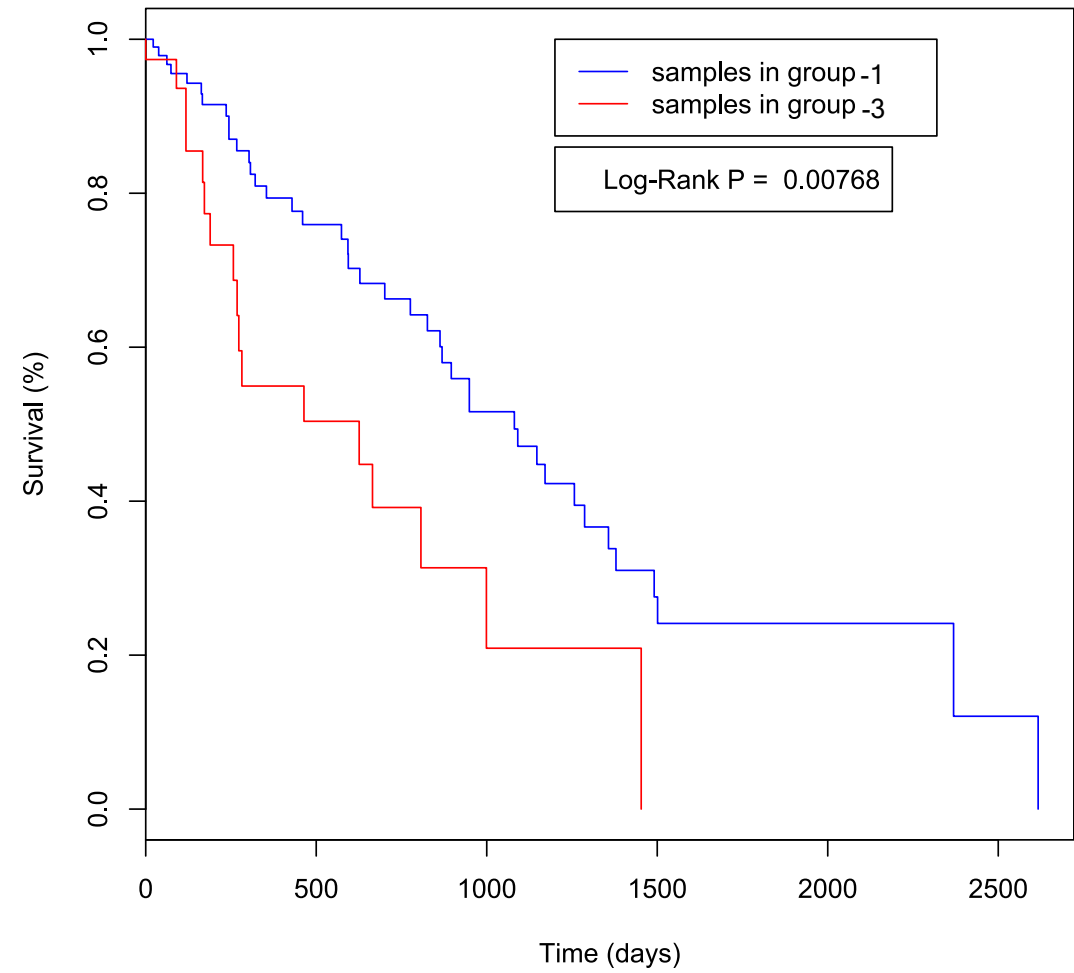

(B)

Figure S10

Supplement: nwy162_Supplemental_Files [file nwy162_supplemental_files.zip › Figure_S10.pdf]

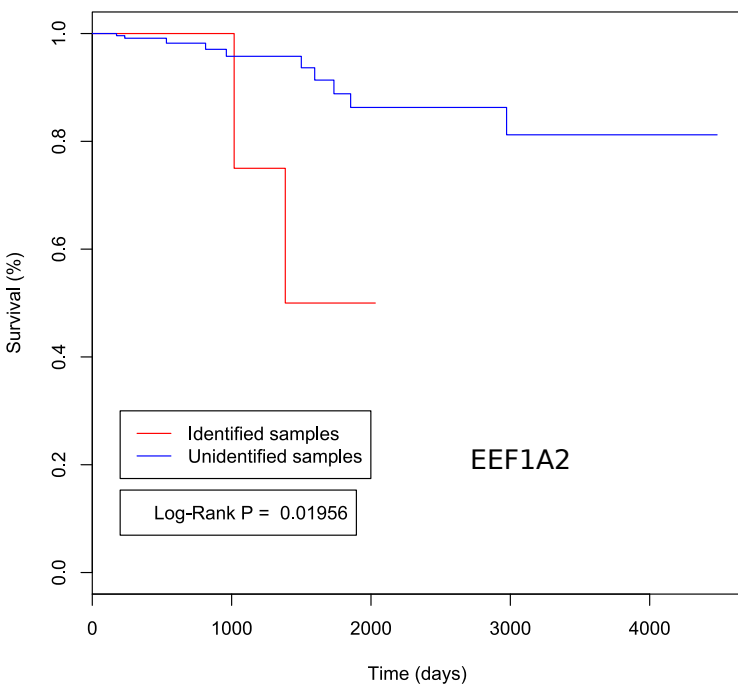

(A)

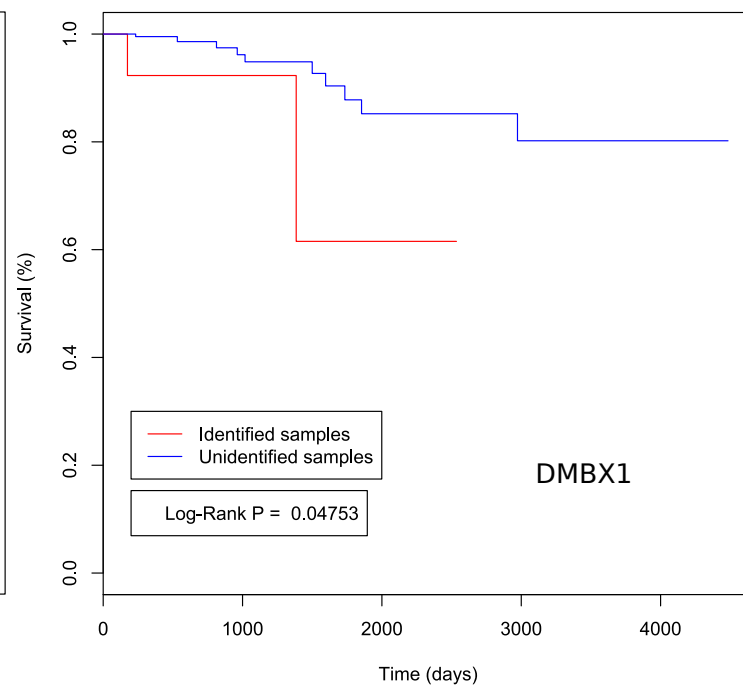

(B)

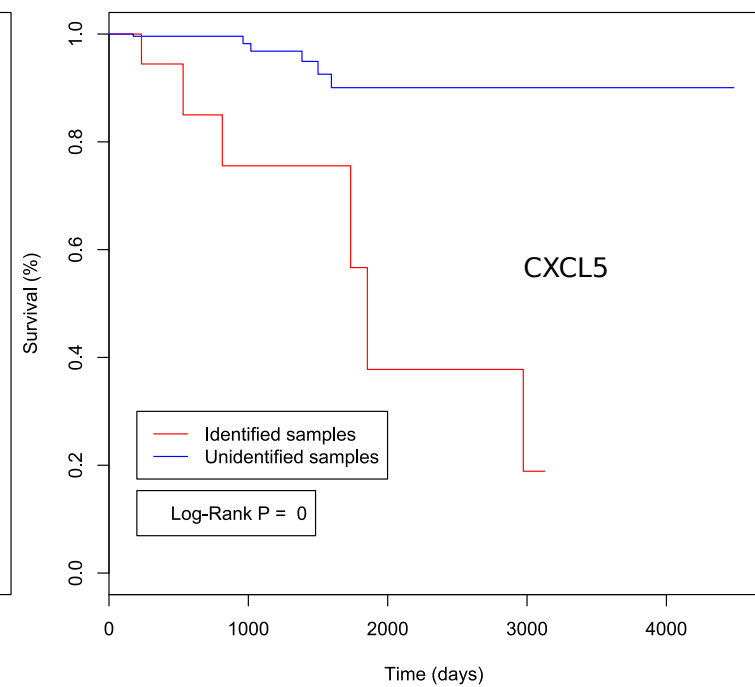

(C)

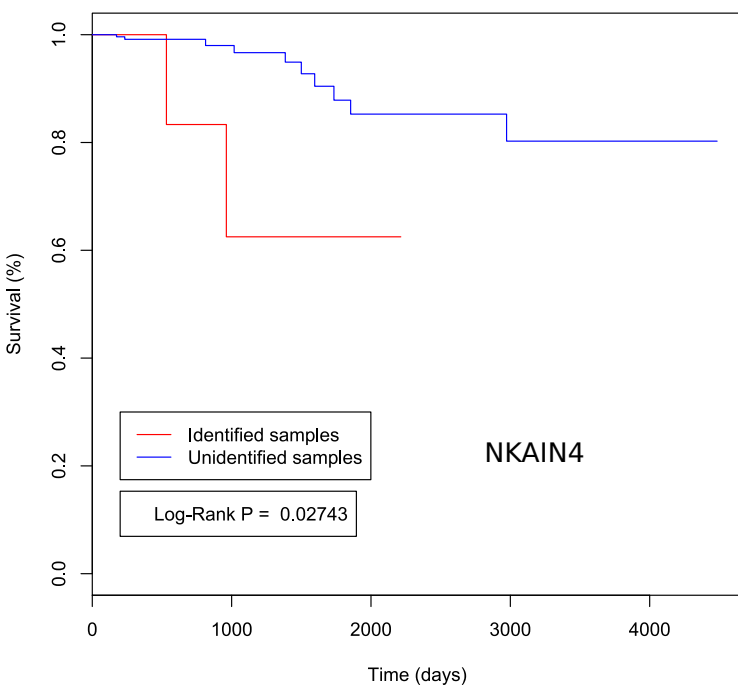

(D)

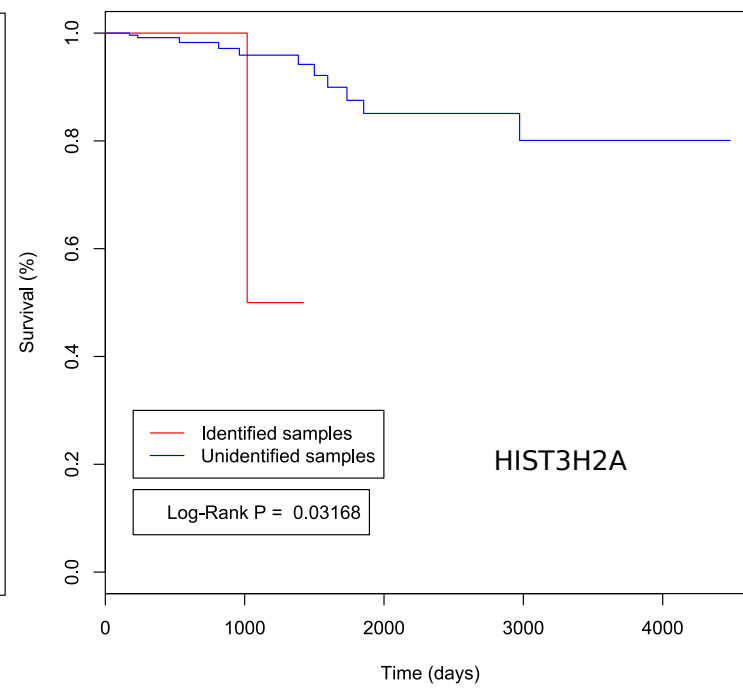

(E)

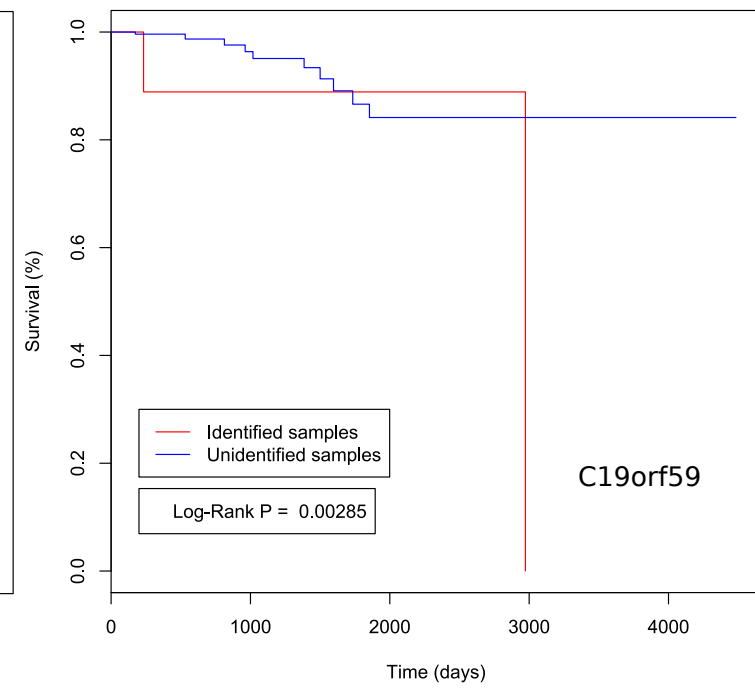

(F)

Figure S11

Supplement: nwy162_Supplemental_Files [file nwy162_supplemental_files.zip › Figure_S11.pdf]

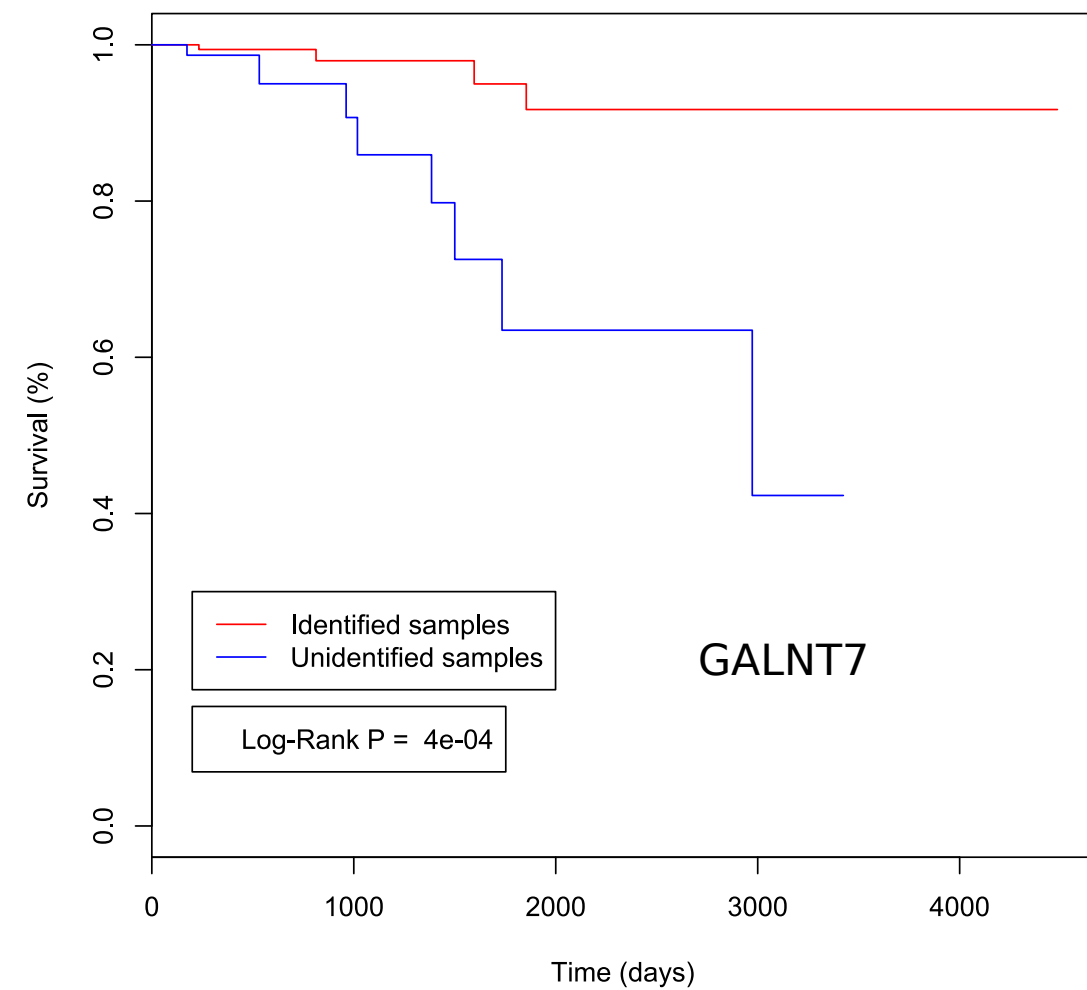

(A)

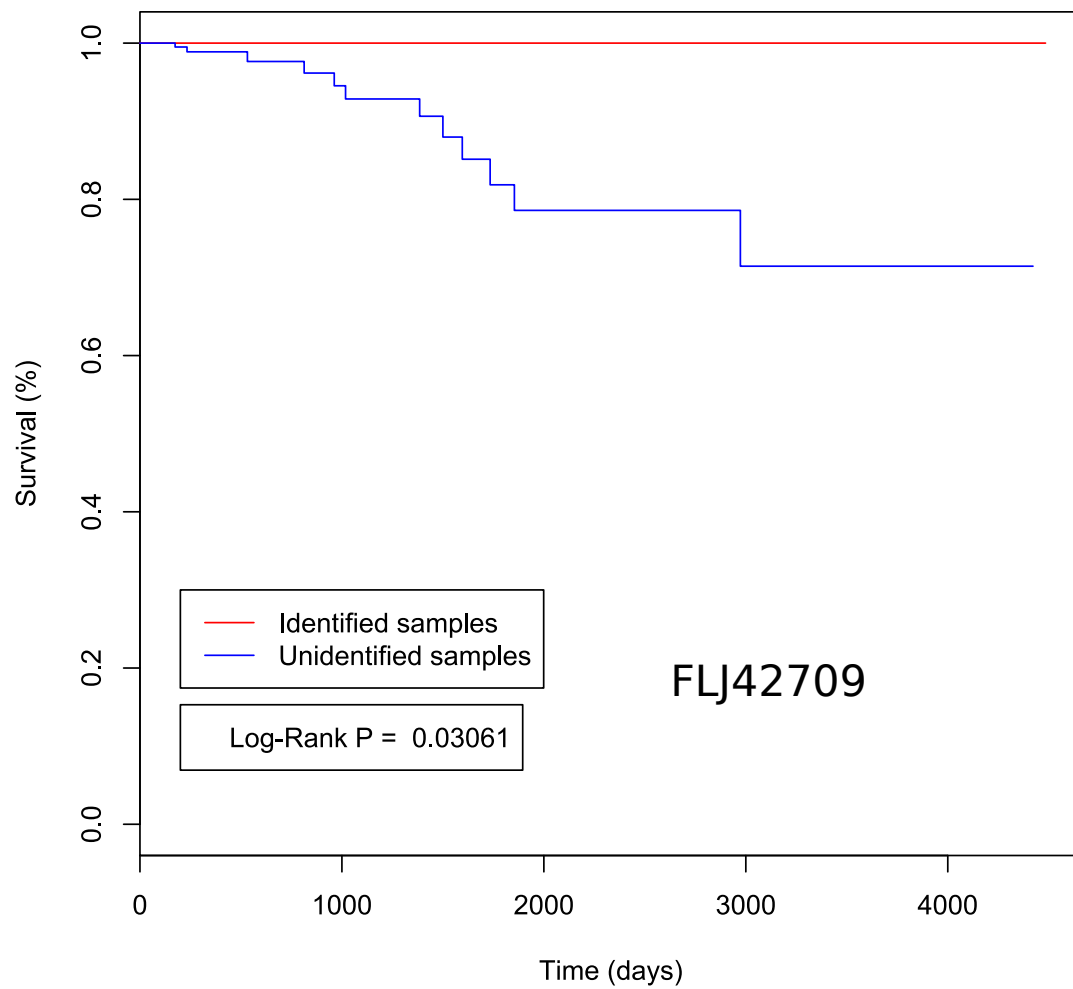

(B)

Figure S12

Supplement: nwy162_Supplemental_Files [file nwy162_supplemental_files.zip › Figure_S12.pdf]

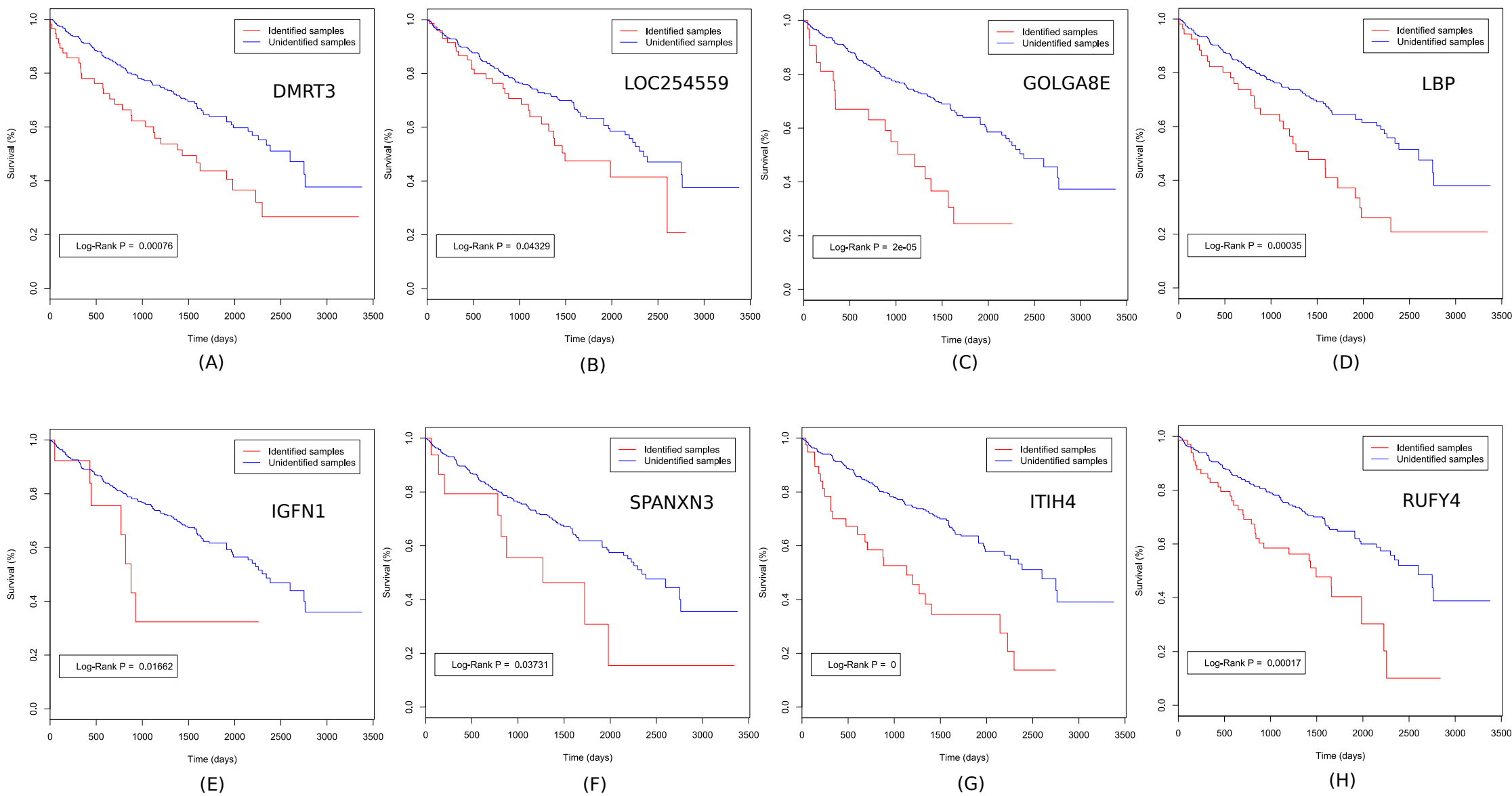

Figure S13

Supplement: nwy162_Supplemental_Files [file nwy162_supplemental_files.zip › Figure_S13.pdf]

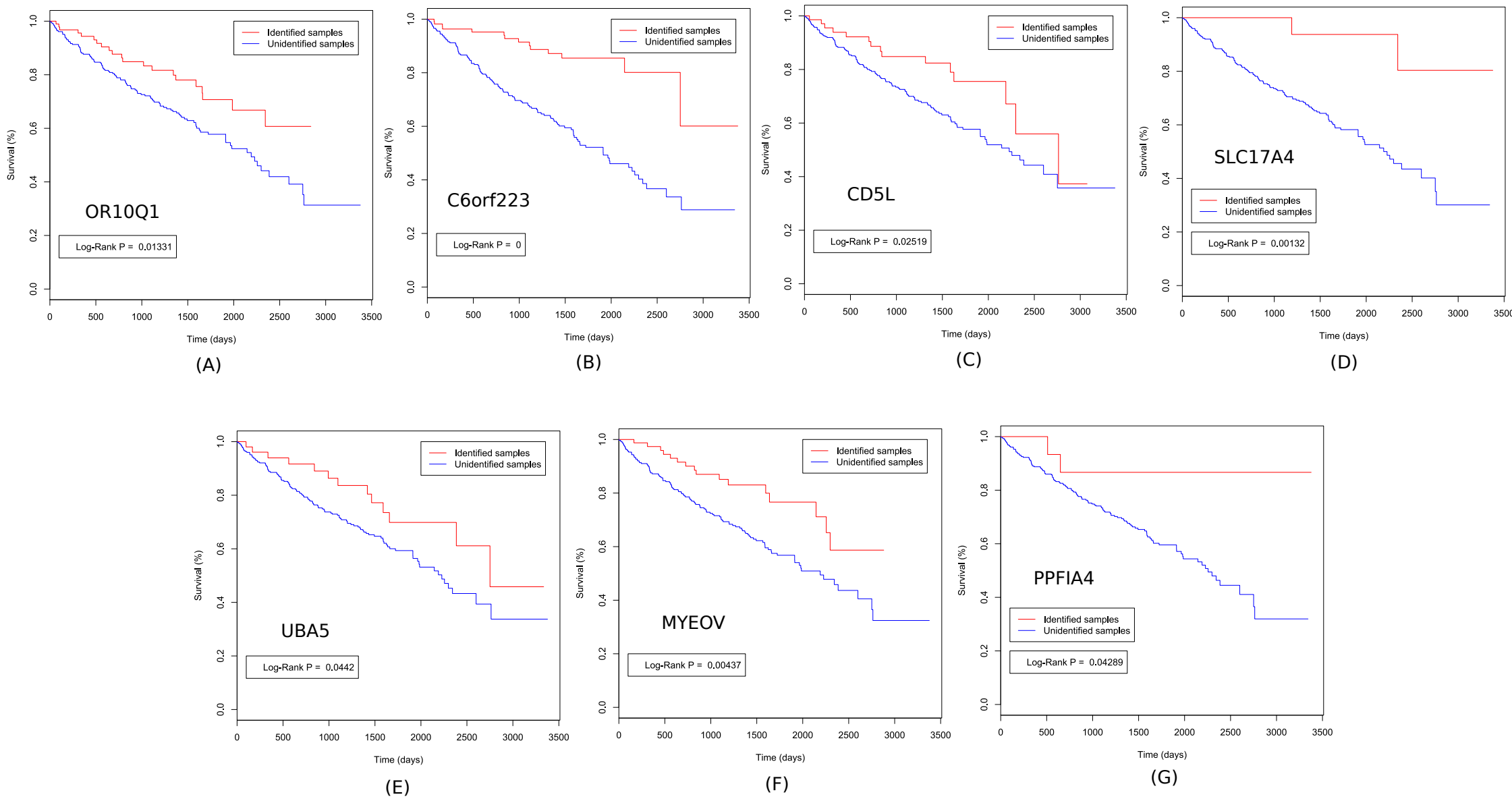

Figure S14

Supplement: nwy162_Supplemental_Files [file nwy162_supplemental_files.zip › Figure_S14.pdf]

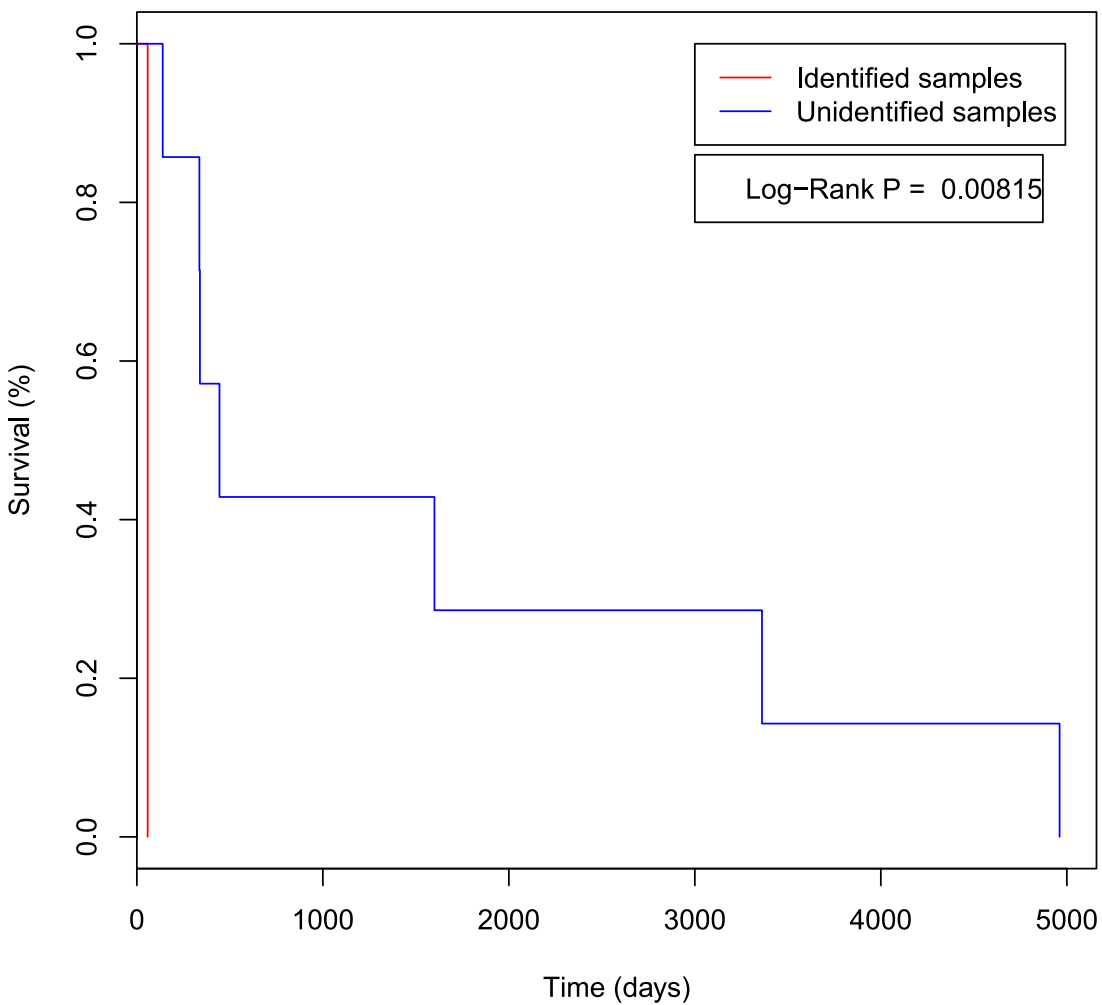

(A)

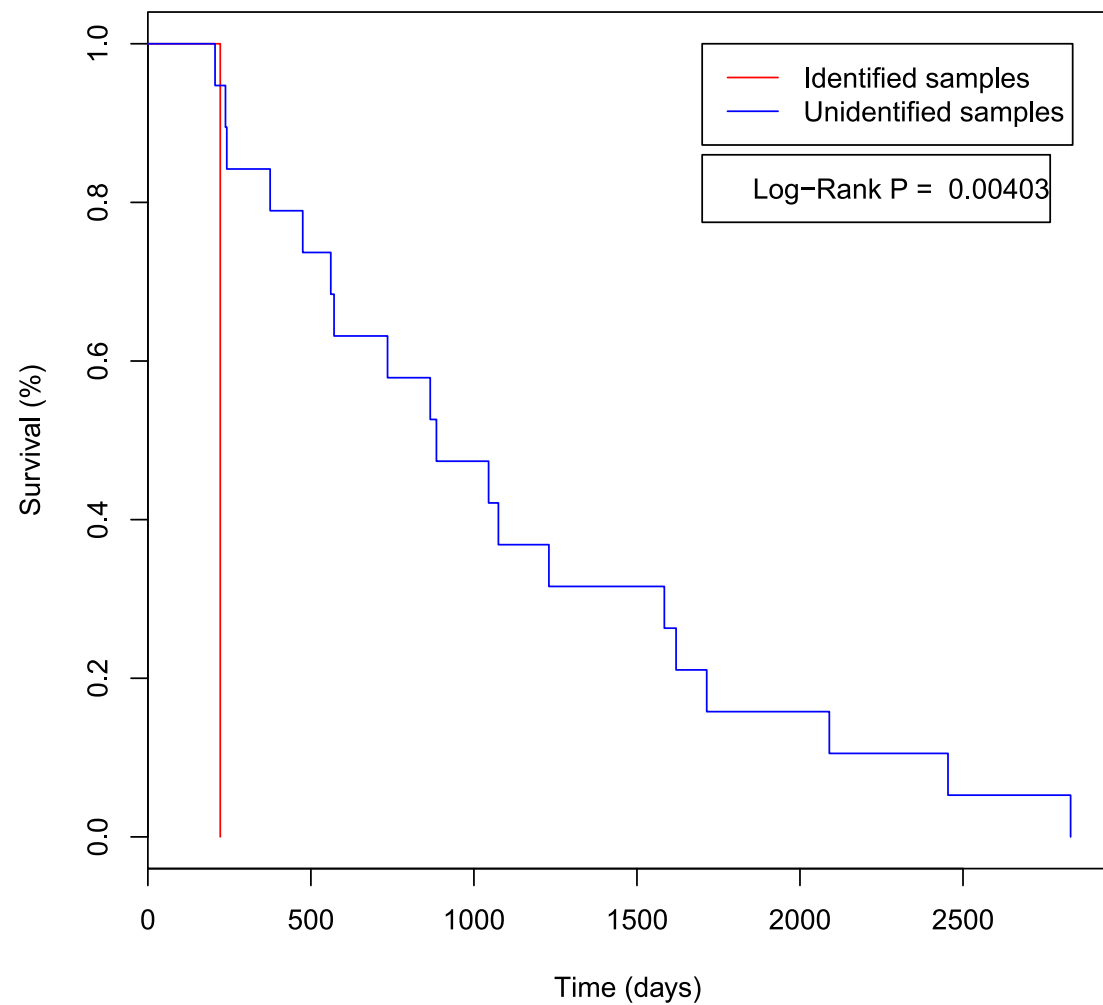

(B)

Figure S15

Supplement: nwy162_Supplemental_Files [file nwy162_supplemental_files.zip › Figure_S15.pdf]

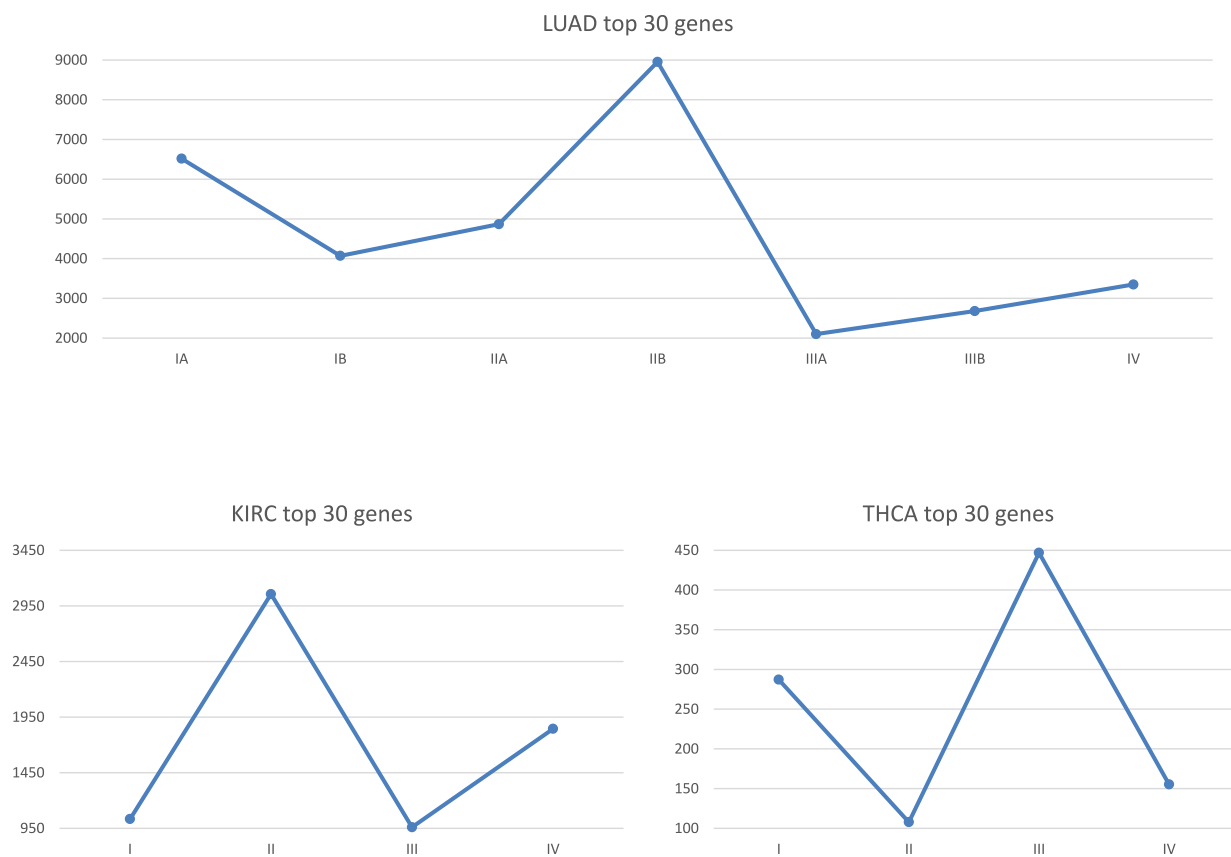

(A)

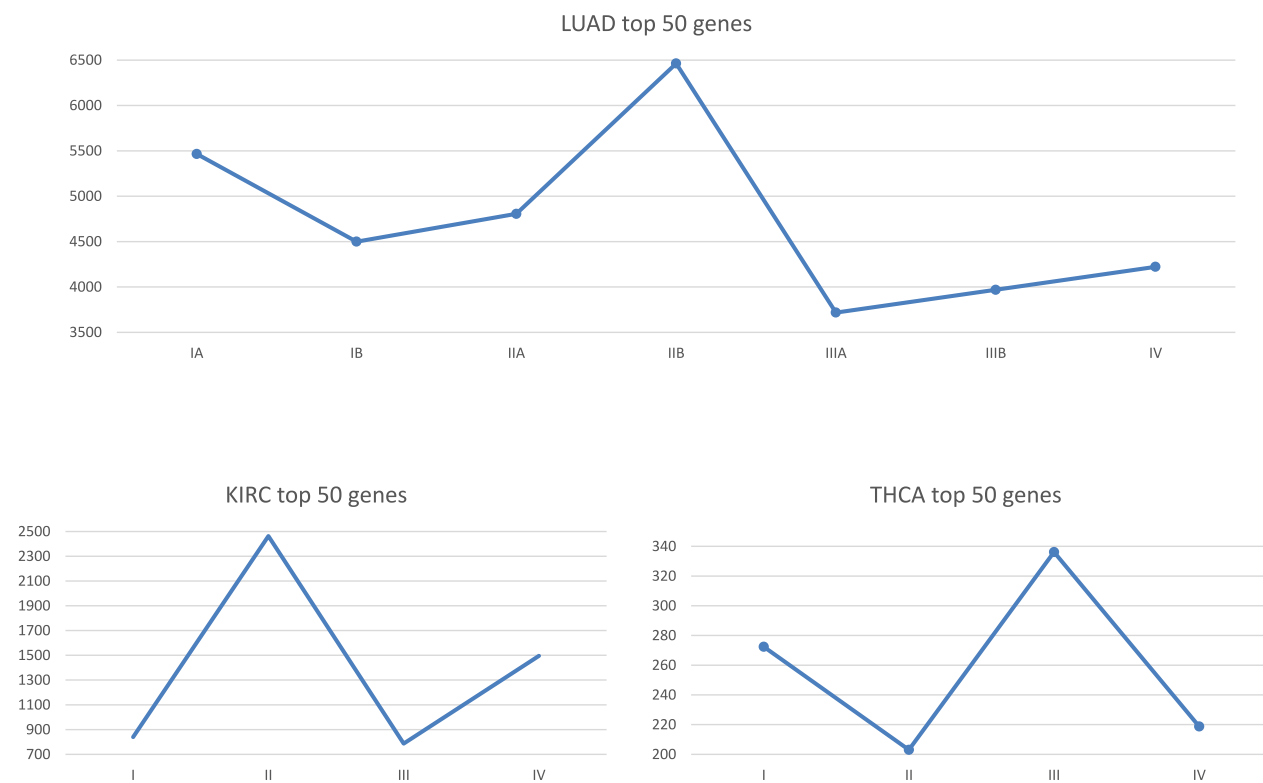

(B)

Figure S16

Supplement: nwy162_Supplemental_Files [file nwy162_supplemental_files.zip › Figure_S16.pdf]

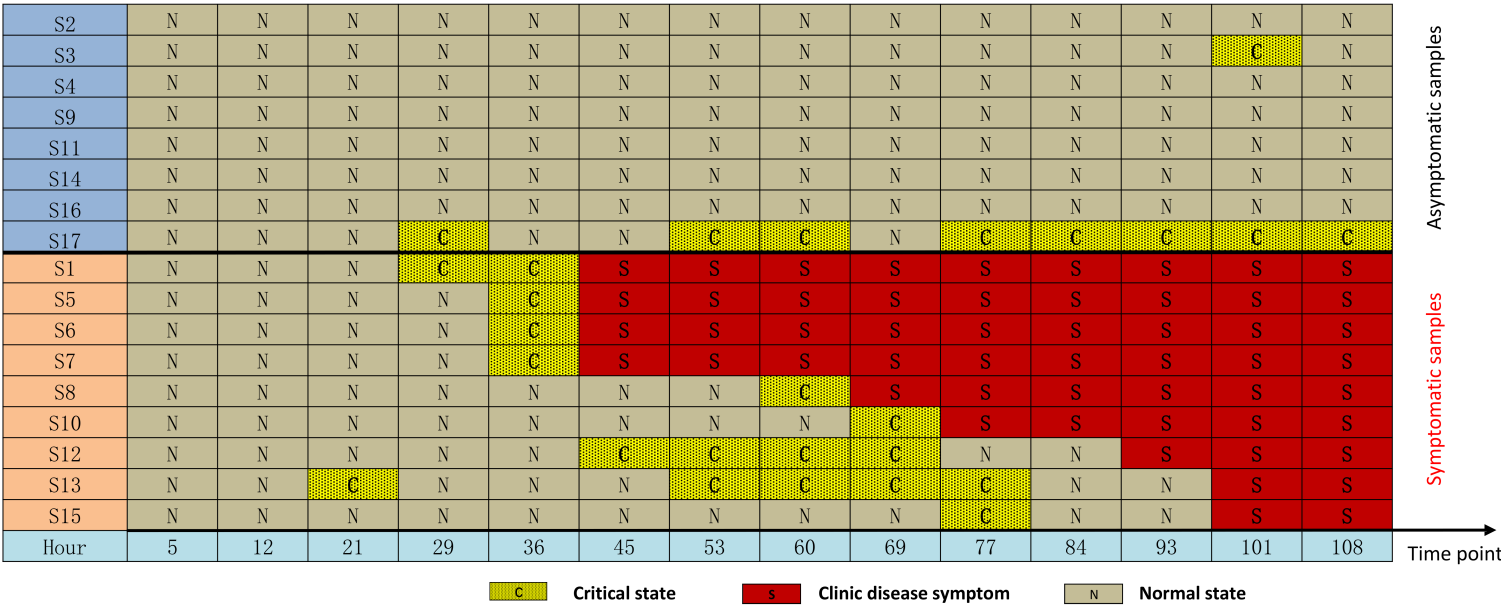

(A)

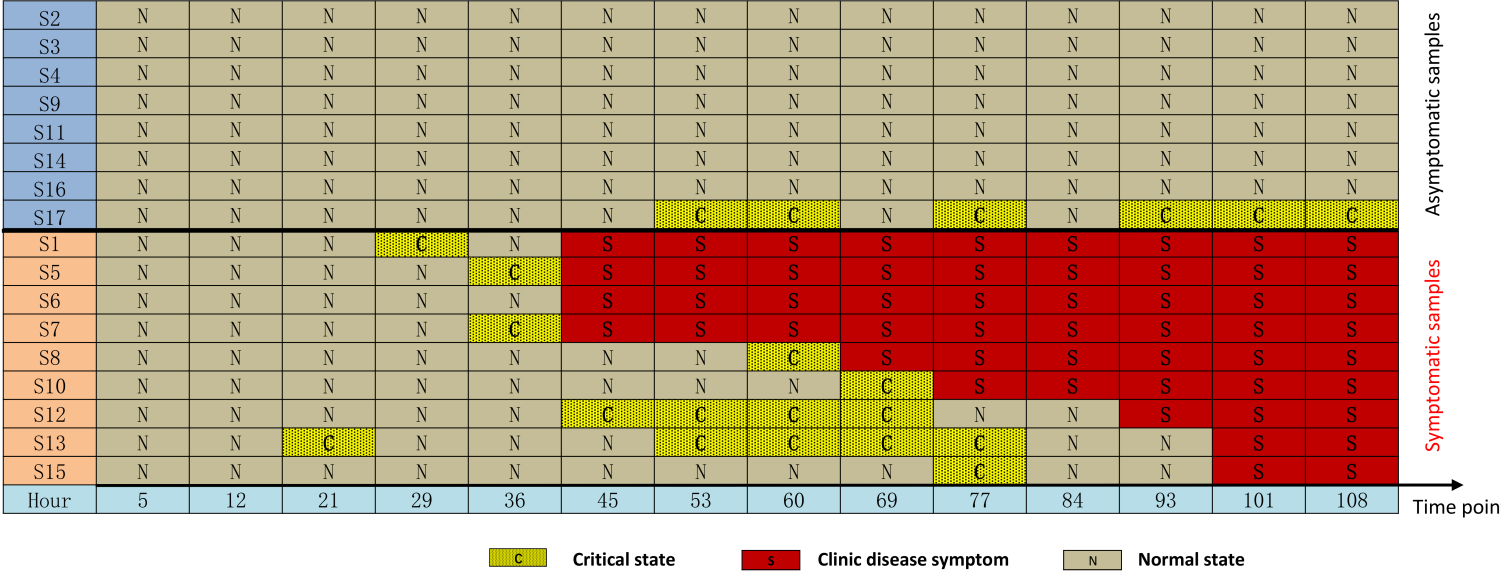

(B)

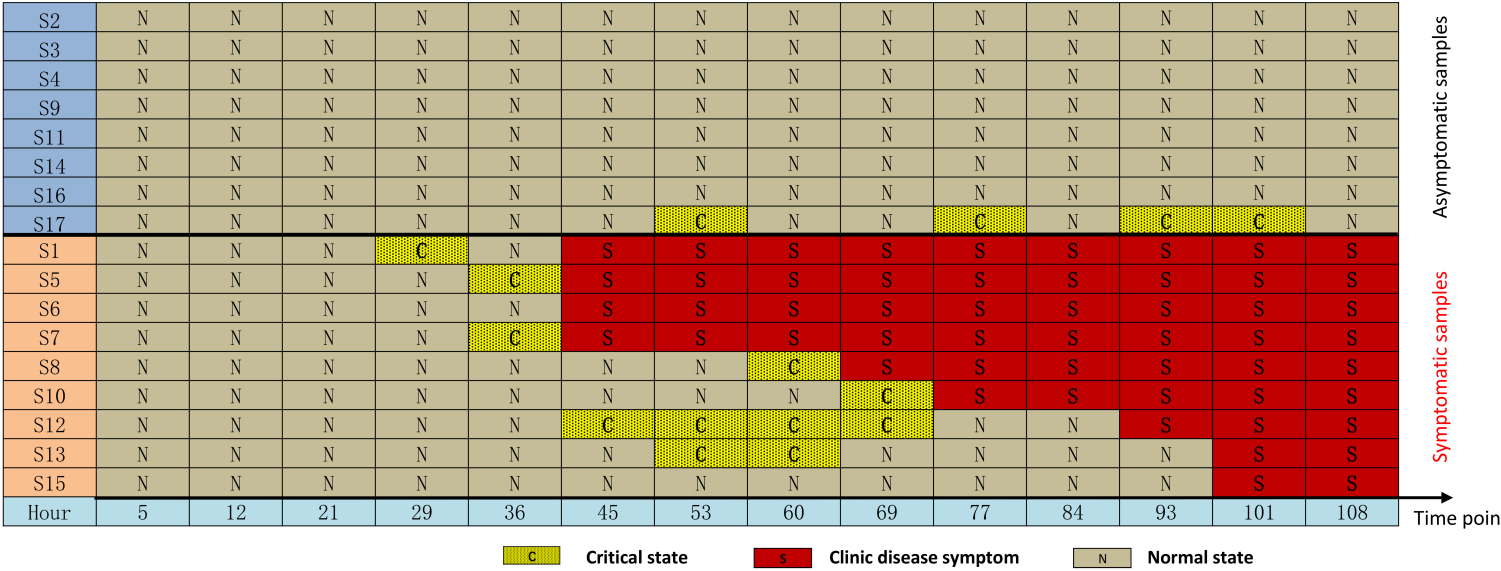

(C)

Figure S17

Supplement: nwy162_Supplemental_Files [file nwy162_supplemental_files.zip › Figure_S17.pdf]

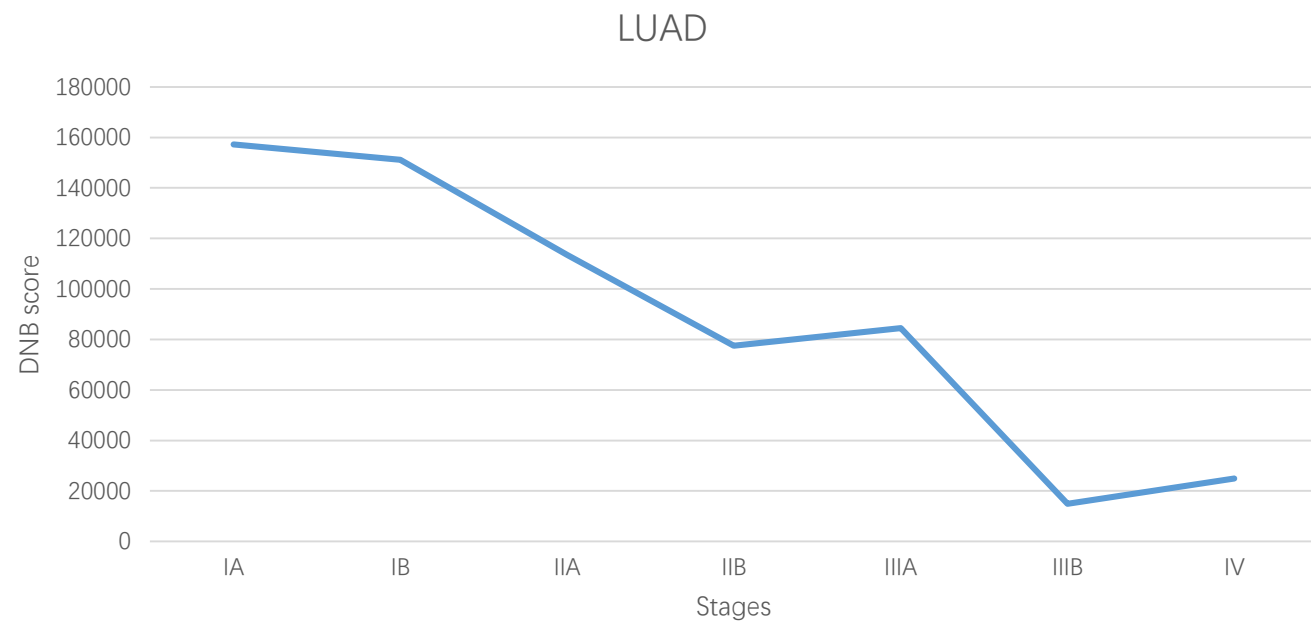

(A)

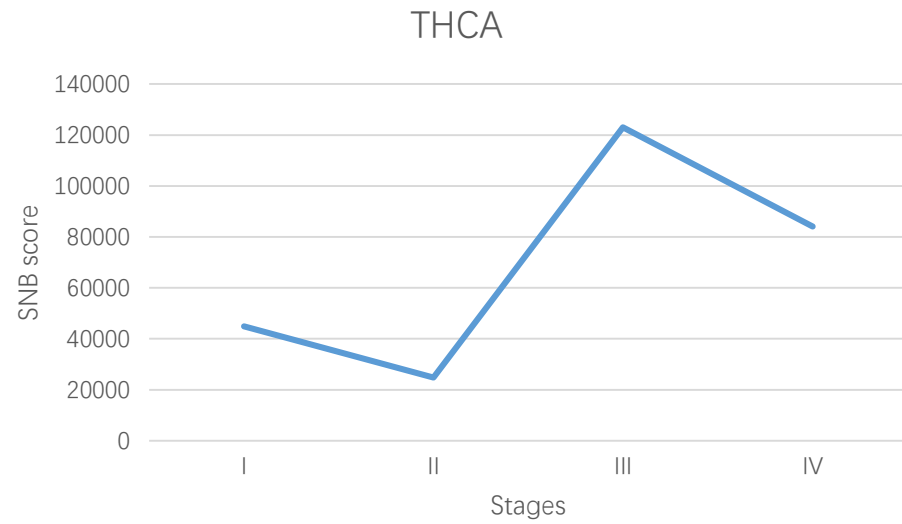

(B)

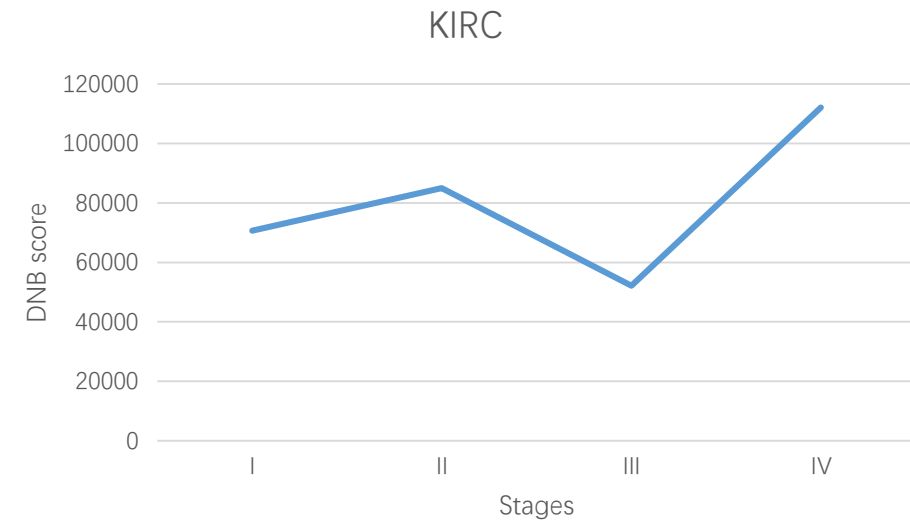

(C)

Figure S18

Supplement: nwy162_Supplemental_Files [file nwy162_supplemental_files.zip › Figure_S18.pdf]

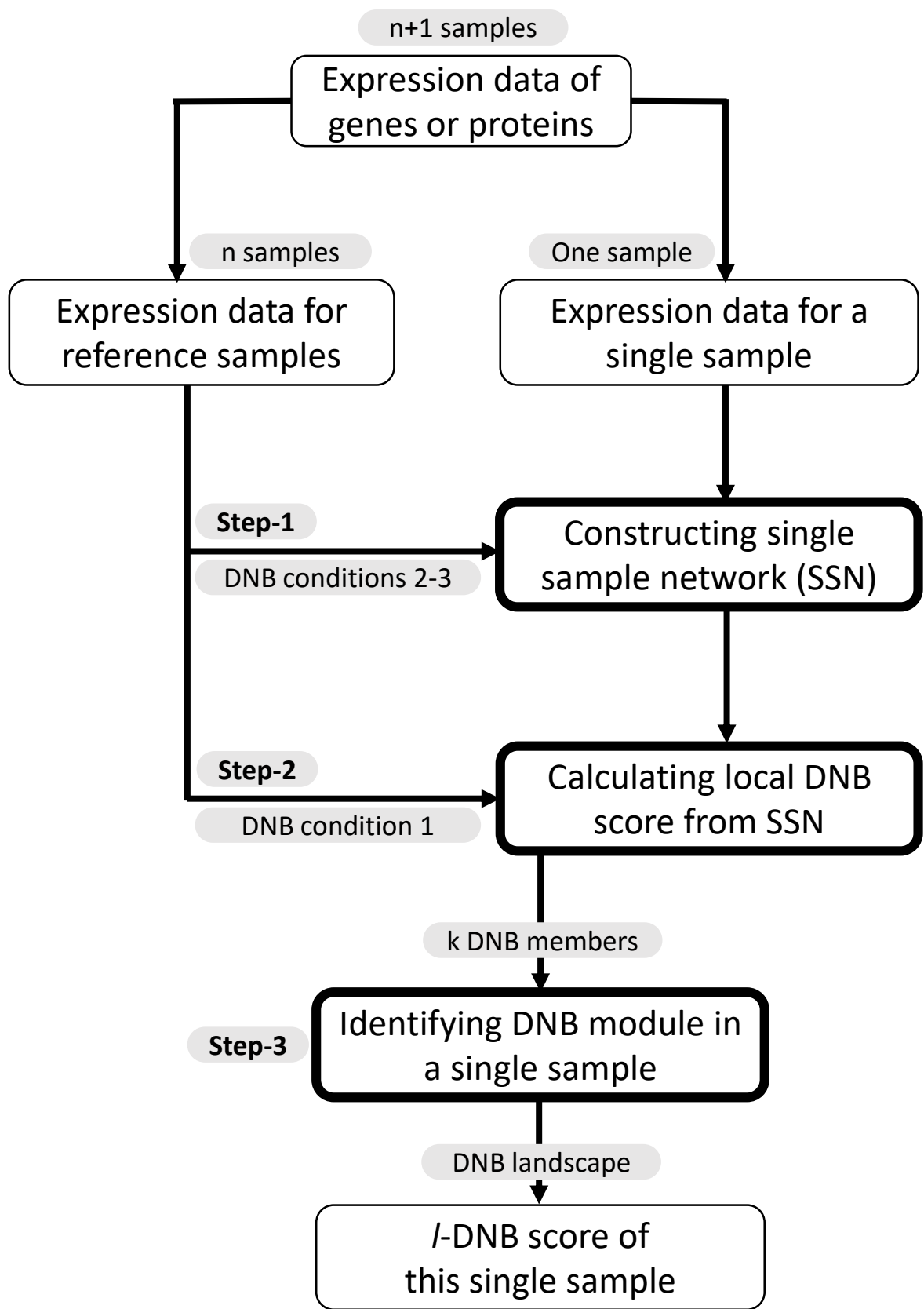

Figure S19

Supplement: nwy162_Supplemental_Files [file nwy162_supplemental_files.zip › Figure_S19.pdf]

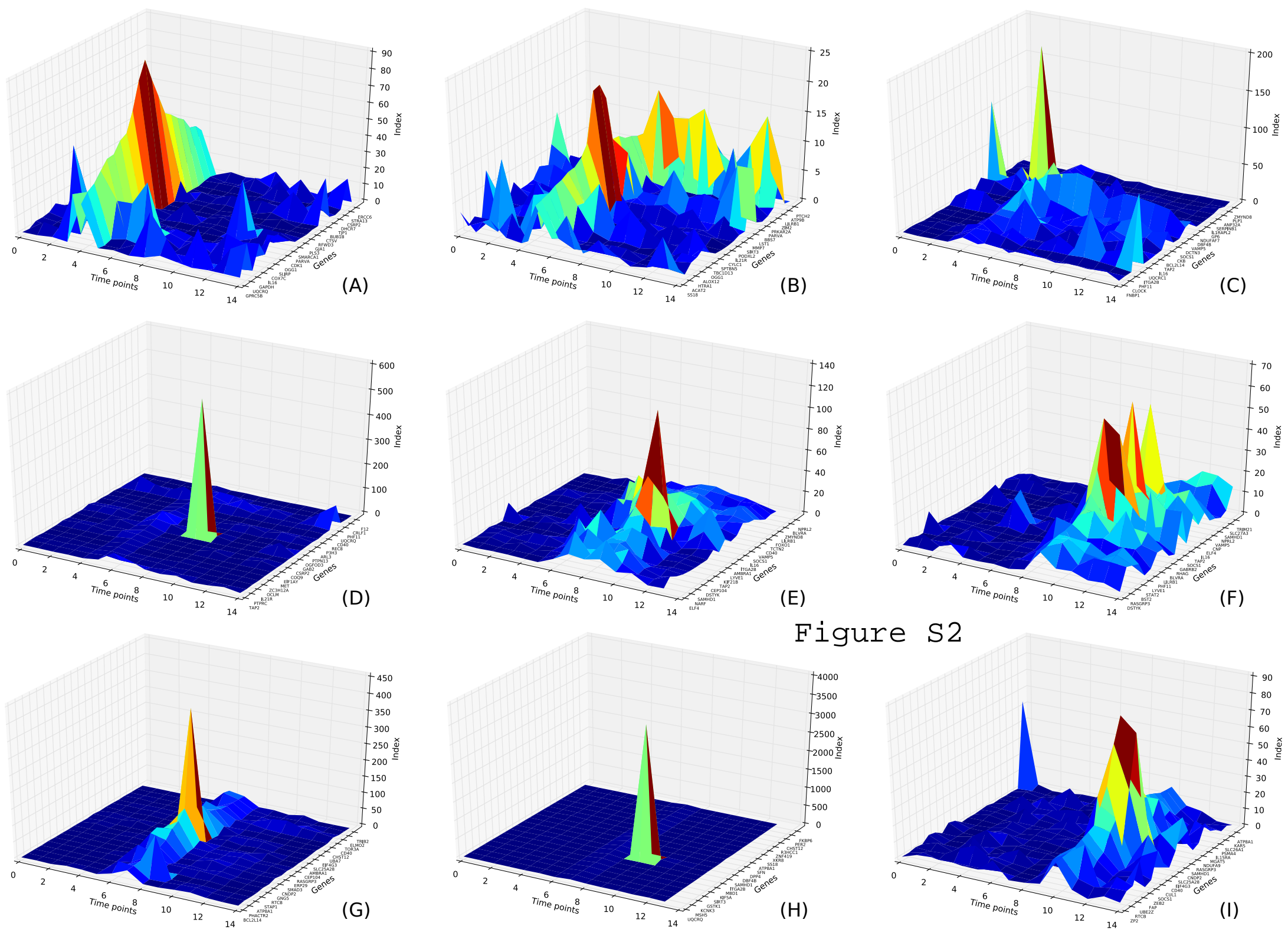

Supplement: nwy162_Supplemental_Files [file nwy162_supplemental_files.zip › Figure_S2.pdf]

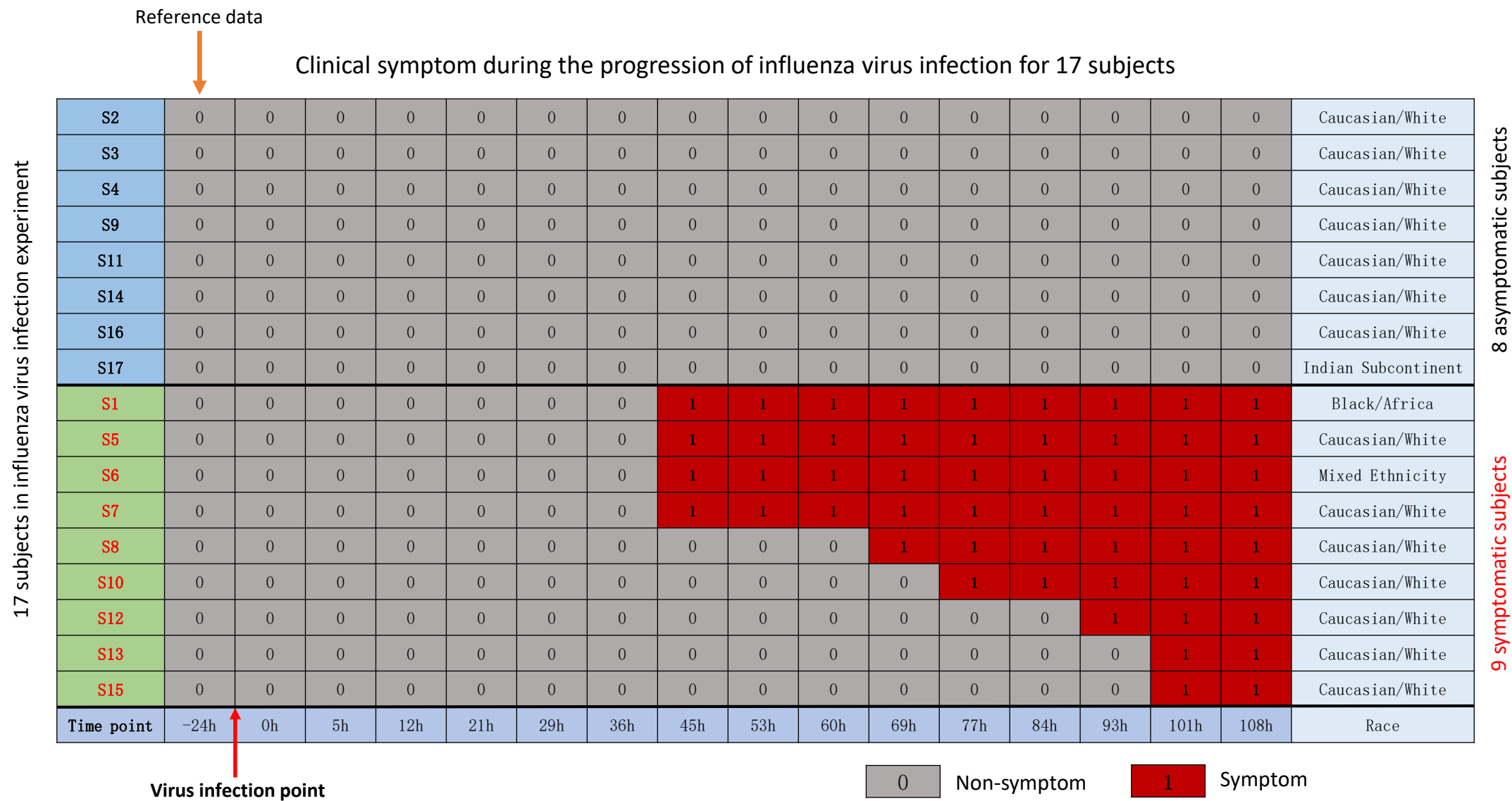

Figure S3

Supplement: nwy162_Supplemental_Files [file nwy162_supplemental_files.zip › Figure_S3.pdf]

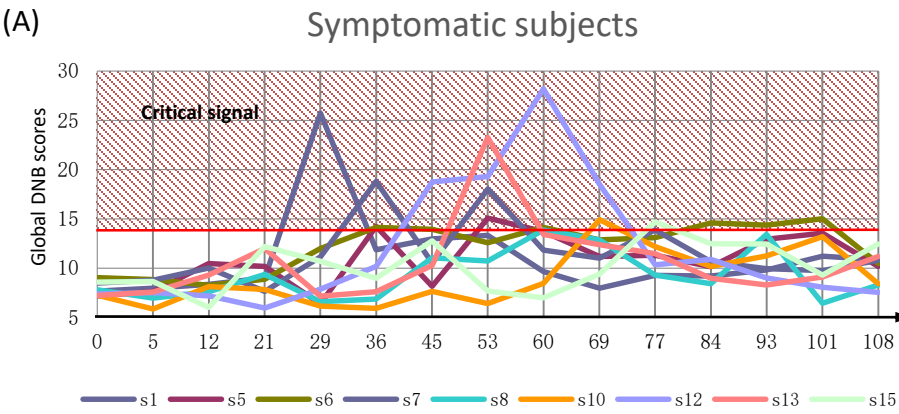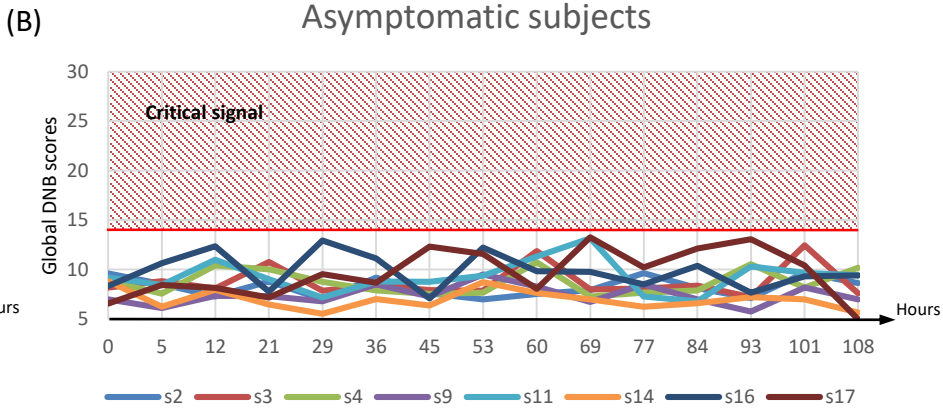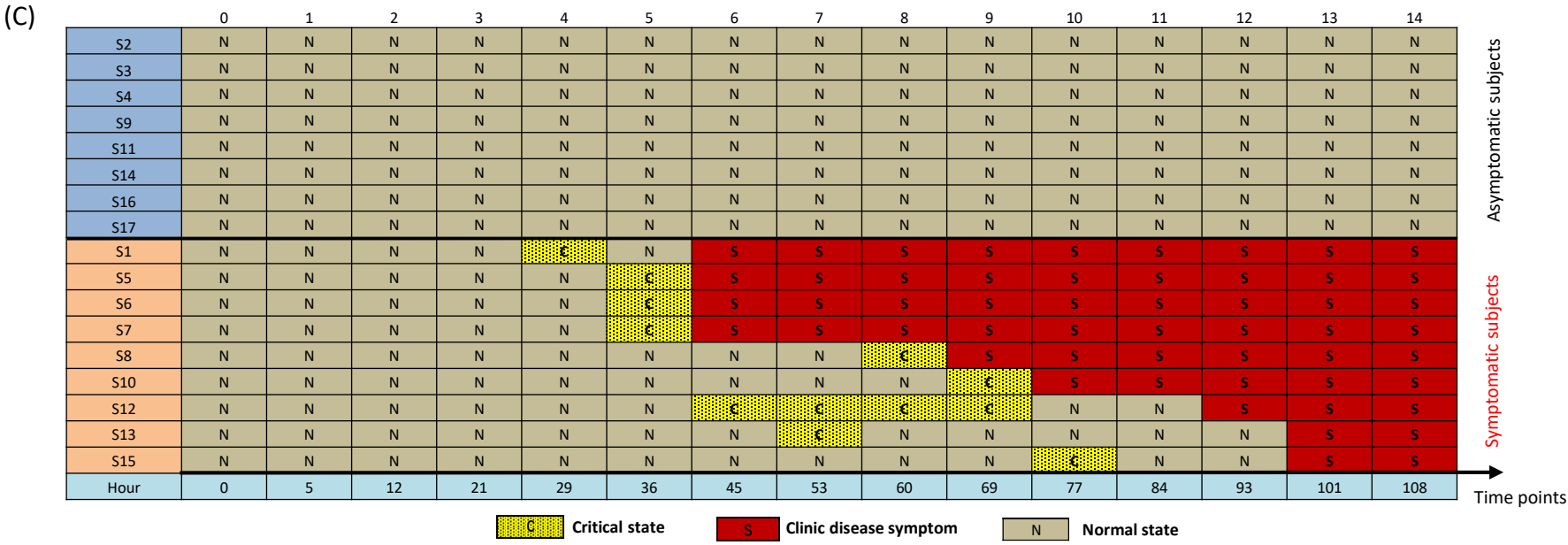

Figure S4

Supplement: nwy162_Supplemental_Files [file nwy162_supplemental_files.zip › Figure_S4.pdf]

$$\text{Threshold} = -0.1752 \cdot k + 19.0887$$

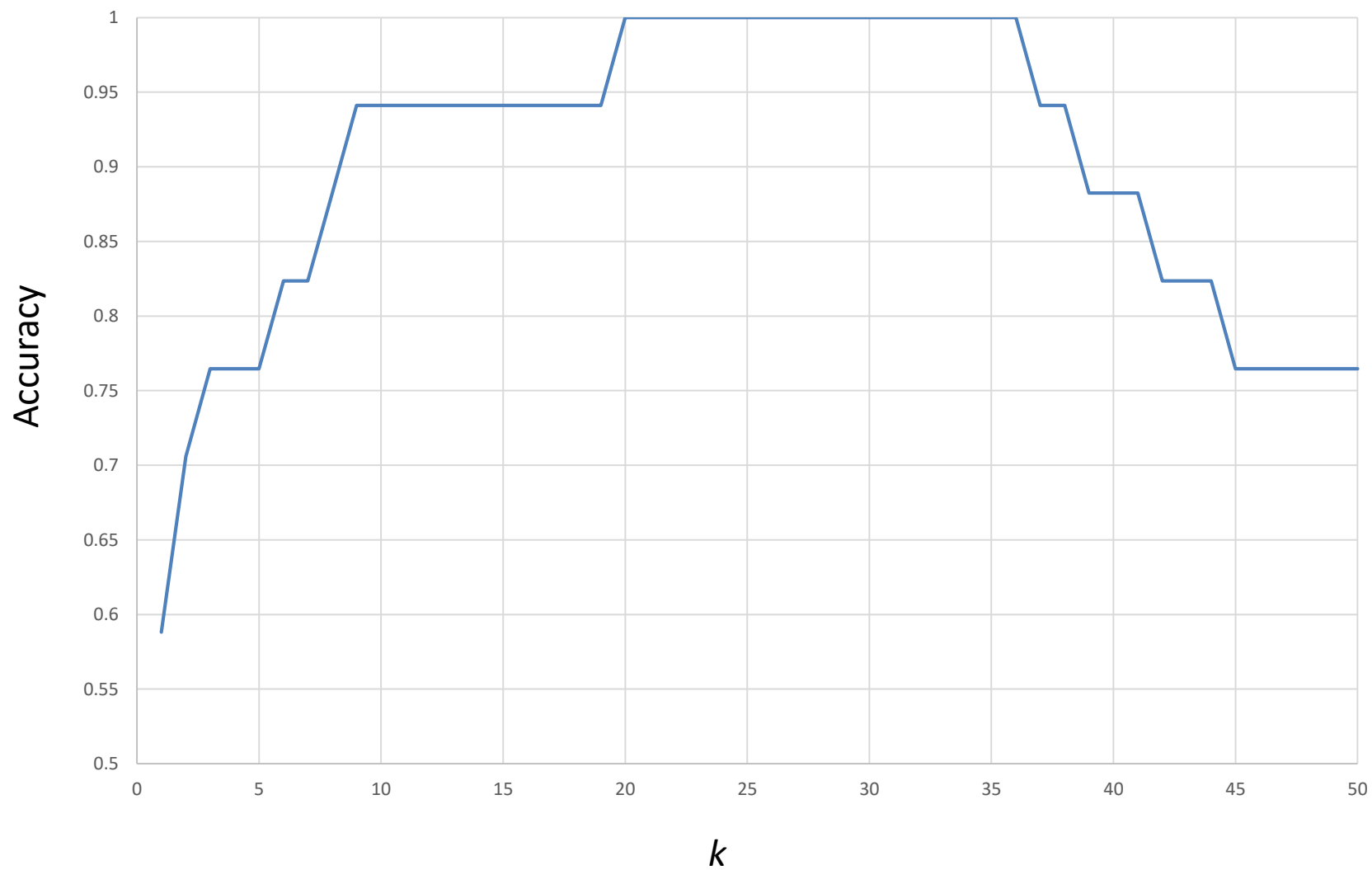

Figure S5

Supplement: nwy162_Supplemental_Files [file nwy162_supplemental_files.zip › Figure_S5.pdf]

## Survival curve for stages IIB and IIIA in LUAD

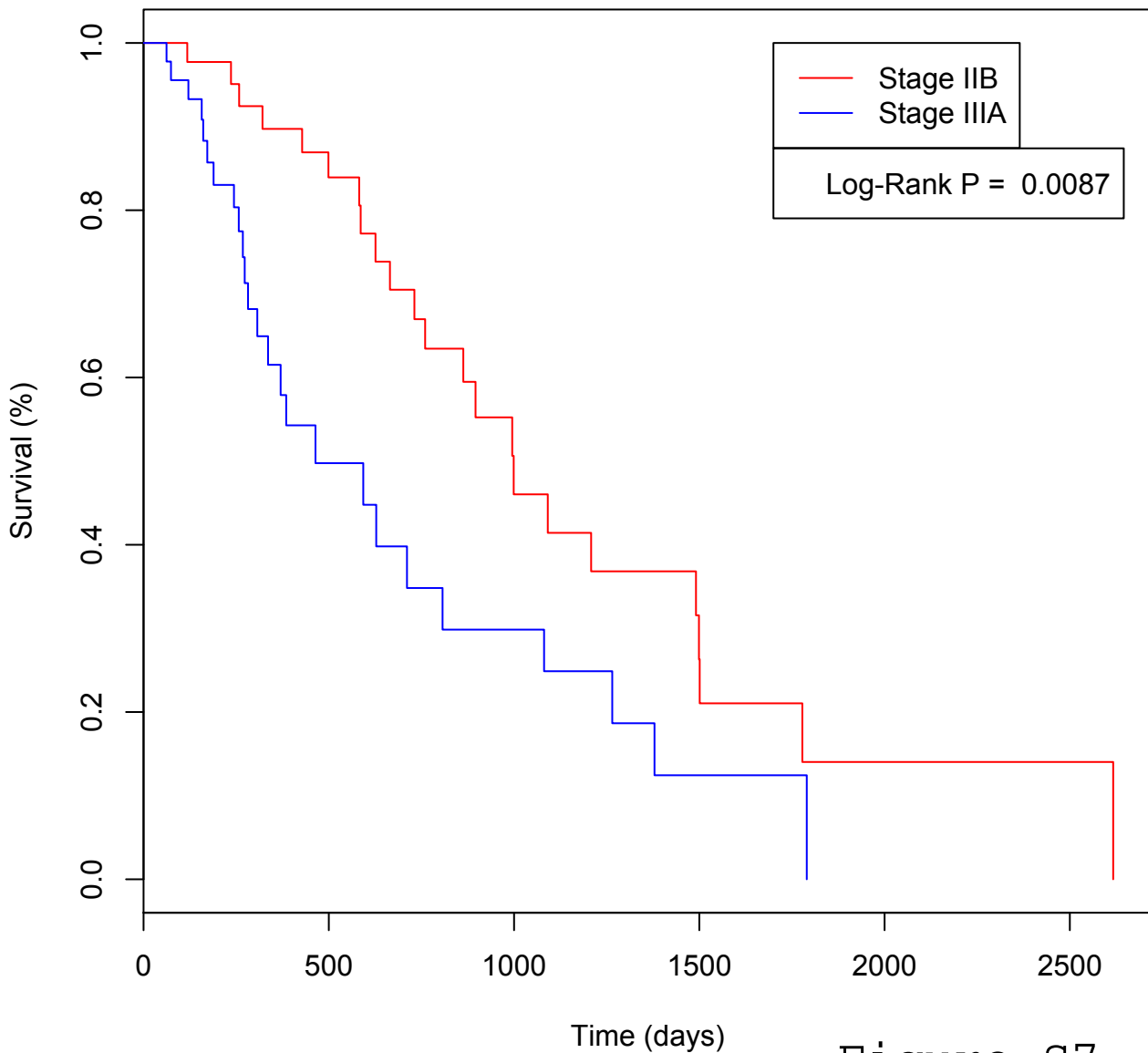

Figure S7

Supplement: nwy162_Supplemental_Files [file nwy162_supplemental_files.zip › Figure_S7.pdf]

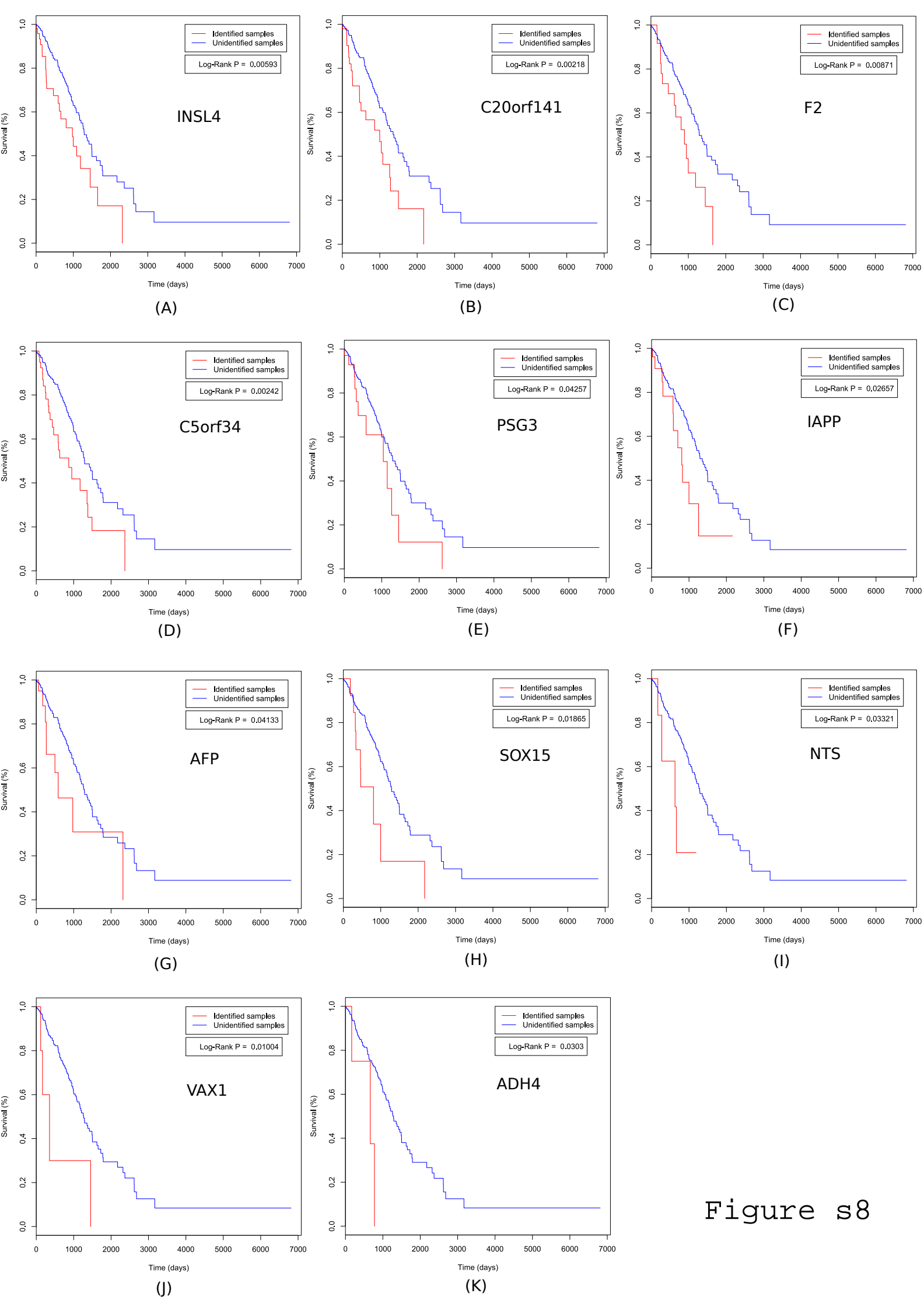

Figure s8

Supplement: nwy162_Supplemental_Files [file nwy162_supplemental_files.zip › Figure_S8.pdf]

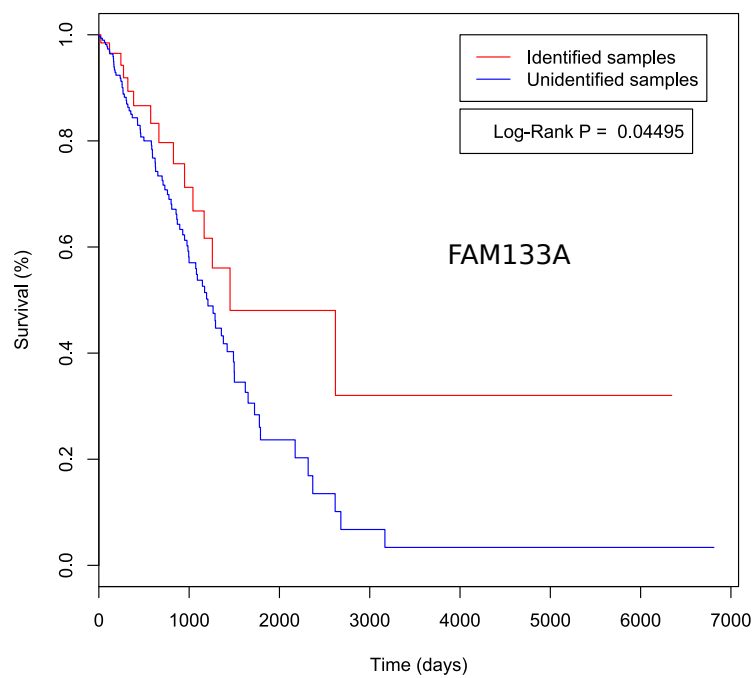

(A)

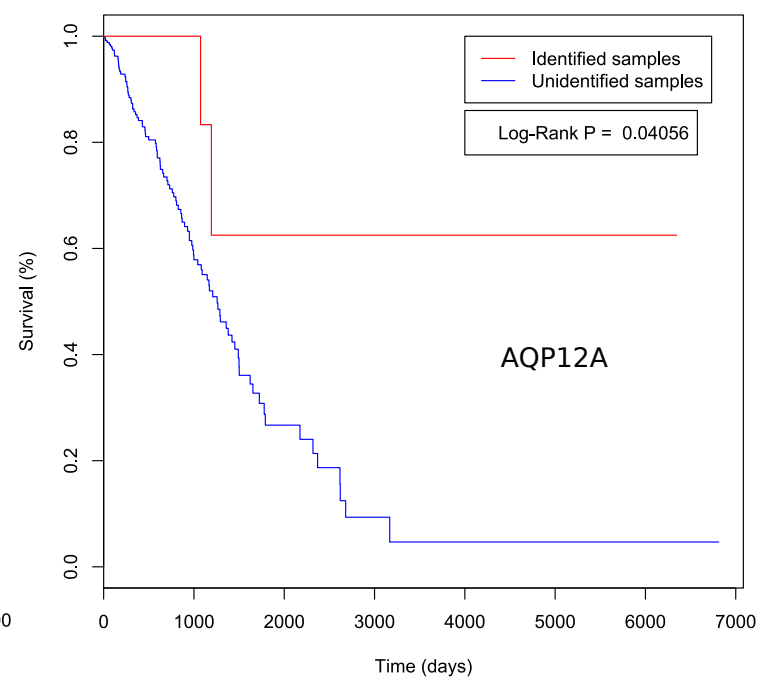

(B)

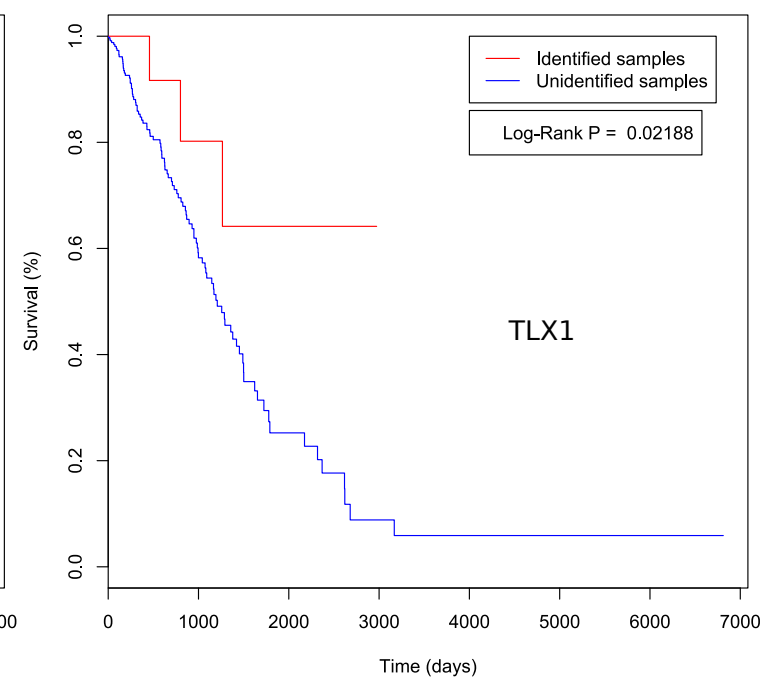

(C)

Figure S9

Supplement: nwy162_Supplemental_Files [file nwy162_supplemental_files.zip › Figure_S9.pdf]
